# Comparison of short- to mid-term outcomes between DAA hemiarthroplasty and PFNA internal fixation in elderly patients with trochanteric fractures: a propensity score-matched retrospective study

**DOI:** 10.3389/fsurg.2026.1954682

**Published:** 2026-10-23

**Authors:** Yanfeng Jia, Liqun Xu, Hongwei Bao, Jingzhao Hou, Zhao Wang

**Affiliations:** Department of Orthopaedic Surgery, Jingjiang People’s Hospital Affiliated to Yangzhou University, Taizhou, Jiangsu, China

**Keywords:** bipolar hemiarthroplasty, direct anterior approach, elderly patients, femoral intertrochanteric fracture, PFNA internal fixation

## Abstract

**Background:**

Trochanteric fractures (TF) are common in elderly patients. Although Proximal Femoral Nail Antirotation (PFNA) internal fixation is widely used, hemiarthroplasty via the direct anterior approach (DAA) has attracted increasing interest because of concerns regarding prolonged bed rest and associated complications. This study compares early outcomes of DAA hemiarthroplasty vs. PFNA internal fixation in elderly TF patients.

**Methods:**

Patients aged ≥75 years with TF treated between January 2018 and January 2024 were retrospectively reviewed. After application of the inclusion and exclusion criteria, 54 patients who underwent DAA hemiarthroplasty were identified. To minimize selection bias, 1:1 propensity score matching was performed based on age, sex, body mass index (BMI), fracture type, Charlson Comorbidity Index (CCI), and American Society of Anesthesiologists (ASA) grade, yielding 54 matched patients who received PFNA. Outcomes included operative duration, blood loss, incision length, bedridden duration, Harris Hip Scores (HHS) at 1, 3, 6, 12, and 24 months, complications, and radiographic findings.

**Results:**

After matching, baseline characteristics were well balanced between the two groups. The PFNA group had significantly less blood loss (220.3 vs. 349.6 mL, *P* < 0.001) and a shorter operative time (40.05 vs. 75.02 min, *P* < 0.001), but a longer total incision length (11.49 vs. 9.29 cm, *P* < 0.001). Bedridden duration was markedly shorter in the DAA group (0.99 vs. 52.86 days, *P* < 0.001). At all follow-up points, HHS was significantly higher in the DAA group (1 month: 78.85 vs. 63.89, *P* < 0.001; 24 months: 92.03 vs. 82.86, *P* < 0.001). Complication rates were lower in the DAA group, although the difference did not reach statistical significance. Mean prosthesis subsidence in the DAA group was 2.7 mm.

**Conclusion:**

In this propensity score-matched retrospective study, DAA hemiarthroplasty was associated with faster early functional recovery and a shorter bedridden duration than PFNA internal fixation in elderly patients with TF, but also with a longer operative time and greater blood loss. The substantial difference in postoperative rehabilitation protocols between the two groups may have contributed to the observed gap in functional outcomes.

**Study design:**

Level of evidence, 3.

## 1 Introduction

Trochanteric fractures (TF) account for about half of all hip fractures, predominantly affecting osteoporotic elderly patients with high mortality (1, 2). The aging population drives a rising TF incidence. TF causes pain, immobility, and inability to stand, severely impairing quality of life. Historically, closed reduction with intramedullary nail fixation—especially Proximal Femoral Nail Antirotation (PFNA)—has been widely used for unstable TF, owing to its minimally invasive nature, good biomechanics, and short operative time (3). However, in osteoporotic elderly patients, PFNA carries elevated risks of fixation failure, screw cutout, varus malunion, and nonunion (4). Even with successful PFNA, prolonged postoperative bed rest worsens prognosis. While not the gold standard, arthroplasty can improve survival and quality of life in selected elderly TF patients (5).

Arthroplasty was traditionally reserved for failed TF fixation, but increasing evidence shows favorable outcomes when used as a primary treatment (6–10). Total hip arthroplasty (THA) offers better durability and may be more suitable for younger or more active patients, whereas hemiarthroplasty is associated with reduces operative time and blood loss, making it preferable for elderly patients without significant acetabular erosion. Accordingly, most arthroplasty patients in our hospital received hemiarthroplasty.

In terms of surgical approach, the posterolateral approach (PLA) has been the most common; however, there has been a notable increase in the number of direct anterior approach (DAA) recently. Previous studies have demonstrated that DAA offers superior early rehabilitation outcomes in the treatment of femoral neck fractures compared to PLA (11–15). These findings suggest that DAA may yield favorable outcomes in treating femoral neck fractures. As an intermuscular surgical pathway, DAA avoids severing the gluteus medius and gluteus minimus muscles, offering potential advantages such as mild postoperative pain, a low dislocation rate, and rapid gait recovery. Nevertheless, few studies have documented the efficacy of DAA arthroplasty in the management of TF. Theoretically, combining the DAA with hemiarthroplasty can offer older patients more stable fixation and earlier functional recovery. Despite the respective strengths and weaknesses of these two surgical modalities, current comparative studies on the efficacy of DAA hemiarthroplasty vs. PFNA internal fixation for TF in the elderly remain relatively scarce.

This study aims to provide a reference for clinical procedure selection by retrospectively comparing and analyzing the clinical efficacy of DAA hemiarthroplasty and PFNA internal fixation in the treatment of TF in elderly patients. Despite these technical challenges, our team has accumulated extensive experience with the DAA for femoral neck fractures. We hypothesized that its muscle-sparing nature could potentially offer accelerated rehabilitation benefits even in the more complex setting of TF.

## 2 Material and methods

### 2.1 Patients

This retrospective study was approved by the Ethics Committee of Jingjiang People's Hospital Affiliated to Yangzhou University. From January 2018 to January 2024, a total of 1,469 consecutive patients with Trochanteric fractures (TF) were treated at our institution. Among them, 1,237 underwent PFNA internal fixation, 44 underwent total hip arthroplasty, and 96 underwent hemiarthroplasty (61 via direct anterior approach [DAA] and 35 via posterolateral approach). The remaining patients received conservative treatment or other internal fixation methods. The inclusion criteria were: (1) unilateral TF; (2) age ≥75 years; (3) treatment with either DAA hemiarthroplasty or PFNA internal fixation. The exclusion criteria were: (1) inability to live independently before injury; (2) pre-existing hip joint disease (e.g., osteoarthritis, rheumatoid arthritis, femoral head necrosis). Of the 61 patients treated with DAA hemiarthroplasty, 7 were excluded before matching: non-compliance with inclusion criteria (*n* = 3), meeting exclusion criteria (*n* = 2), loss to follow-up (*n* = 1), incomplete or missing data (*n* = 1). The remaining 54 patients with complete follow-up data were included. Missing follow-up data were handled by exclusion before propensity score matching. From the initial pool of 1,237 patients treated with PFNA, a total of 892 patients met the same inclusion and exclusion criteria (Figure 1).

**Figure 1:**
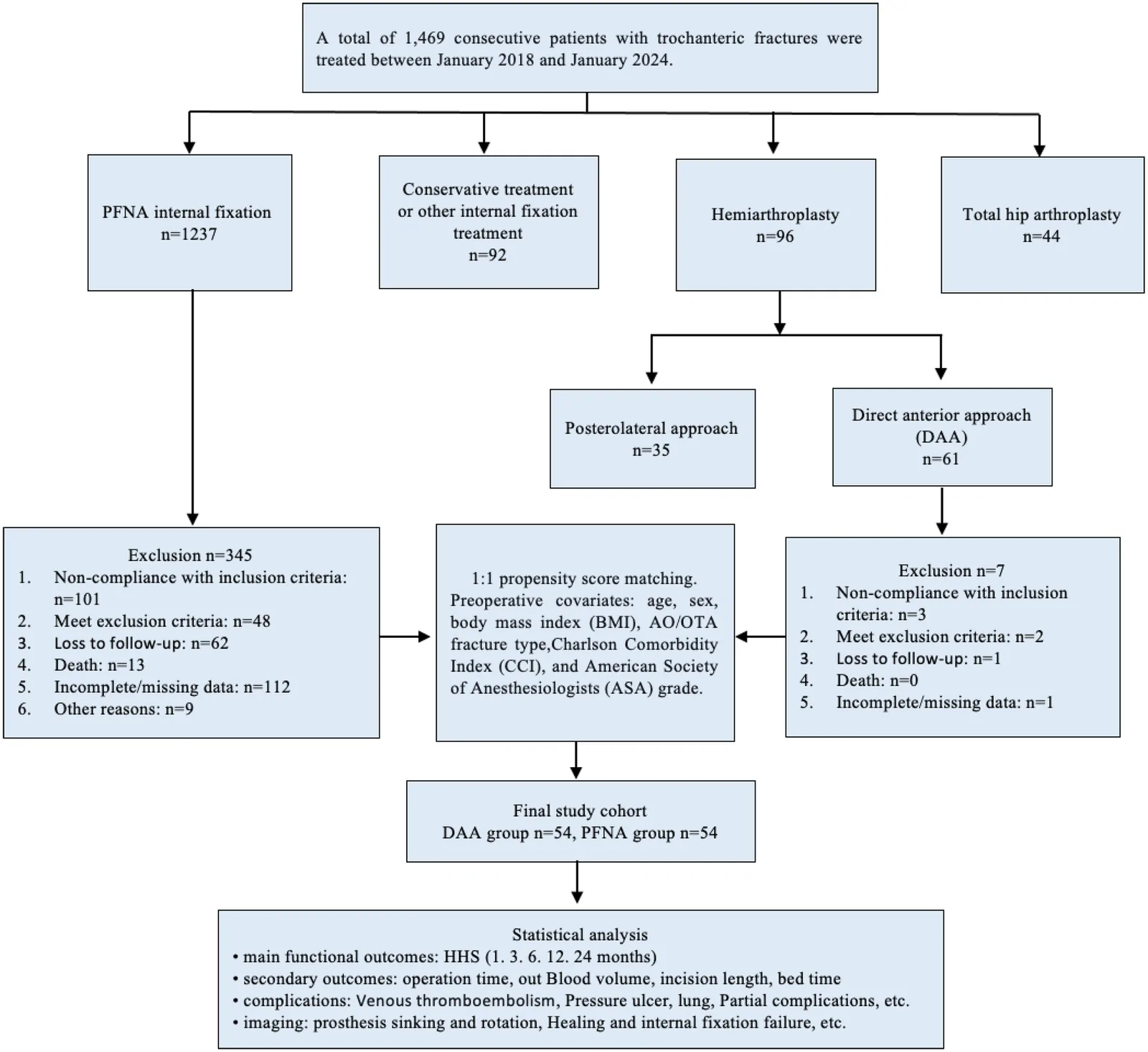
Patient flow diagram.

To minimize selection bias, 1:1 propensity score matching (PSM) was performed using a logistic regression model. The propensity score for each patient was calculated based on the following preoperative covariates: age, sex, body mass index (BMI), fracture type classified according to the AO/OTA system (all included fractures were AO/OTA 31-A fractures), Charlson Comorbidity Index (CCI), and American Society of Anesthesiologists (ASA) grade (16). Unmeasured confounders may persist. Nearest-neighbor matching was applied with a caliper width of 0.2 times the standard deviation of the logit of the propensity score. This process matched 54 patients treated with PFNA to 54 patients treated with DAA, yielding a final study cohort of 108 patients (54 per group). Treatment selection was not randomized. The choice between DAA hemiarthroplasty and PFNA was made by the treating surgeon in consultation with the patient and family and was influenced by medical comorbidity, fracture pattern, bone quality, pre-injury mobility, expected tolerance of bed rest, and patient preference. This represents a potential source of selection bias.

The sample size was determined by the number of eligible patients after propensity score matching rather than by an priori power calculation. *Post hoc* power analysis was performed using the observed matched sample size (54 patients per group) and the observed effect estimates.

Baseline characteristics after matching were well balanced between the two groups (Table 1). In the DAA group, a long-stem femoral component was used in all patients (51 cementless stems and 3 cemented stems). A bipolar head was used in all patients. Intraoperative fixation of the greater or lesser trochanter was performed in 53 patients, while one patient did not require fixation due to an old fracture with a stable femoral component. In the PFNA group, closed reduction was achieved in 53 of the 54 matched patients, with only one case requiring open reduction.

**Table 1:** Baseline demographic and clinical characteristics of the matched cohort.

Characteristic	DAA group	PFNA group	*P* value
Number of patients (*n*)	54	54	—
Sex (female/male)	31/23	33/21	—
Age	81.17 ± 3.92	81.58 ± 3.46	0.558
BMI (kg/m^2^)	23.31 ± 2.67	23.51 ± 2.06	0.659
AO/OTA fracture type, *n* (%)			0.721
31-A1 (stable)	18 (33.3%)	17 (31.5%)	
31-A2 (unstable)	28 (51.9%)	29 (53.7%)	
31-A3 (reverse oblique)	8 (14.8%)	8 (14.8%)	
CCI	2.1 ± 1.2	2.0 ± 1.1	0.654
ASA grade, *n* (%)			0.592
II	15 (27.8%)	16 (29.6%)	
III	34 (63.0%)	33 (61.1%)	
IV	5 (9.3%)	5 (9.3%)	

SD, standard deviation; BMI, body mass index; AO/OTA, Arbeitsgemeinschaft für Osteosynthesefragen/Orthopaedic Trauma Association; ASA, American Society of Anesthesiologists; CCI, Charlson Comorbidity Index.

### 2.2 Data collection

This study documented the intraoperative findings and the 2-year postoperative follow-up results for all subjects. Data collection was conducted collaboratively by two authors. Intraoperative data encompassed the duration of the operation, blood loss volume, and incision length. Postoperative data were categorized into functional outcomes and imaging findings. Functional outcomes included postoperative bed rest duration, Harris Hip Scores (HHS), complications, and rehabilitation outcomes, whereas imaging findings were obtained from measurements on x-ray images. Relevant data were documented at 1 month, 3 months, 6 months, 12 months, and 24 months postoperatively. HHS and radiographic assessments were performed by independent assessors blinded to treatment group. Perioperative mortality during hospitalization and mortality during the 2-year follow-up period were recorded. Pulmonary complications were defined as pneumonia, respiratory failure, acute exacerbation of chronic obstructive pulmonary disease, or pleural effusion. Venous thrombosis was diagnosed by Doppler ultrasonography, which was performed systematically in all patients before discharge. All patients received low-molecular-weight heparin for 7 days after surgery, combined with early mobilization and intermittent pneumatic compression.

### 2.3 Surgical techniques

Both surgical procedures were performed by four experienced surgeons at our hospital.

DAA hemiarthroplasty: The patient was positioned supine. An approximately 8–10 cm incision was made proximally along the line connecting the anterior superior iliac spine and the lateral femoral condyle. After incising the skin and subcutaneous tissue, the fascia of the tensor fasciae latae was cut and reflected medially toward the space of the rectus femoris. The branch of the lateral femoral circumflex artery was ligated. A specifically designed acetabular retractor was used to expose the anterolateral joint capsule, which was then incised for osteotomy. The femoral head was excised, and the capsule was released. The operating table was flexed to position the lower limb in hyperextension, adduction, and external rotation, thereby adequately exposing the proximal femur. The greater or lesser trochanter was reconstructed and fixed with steel wires or titanium cables in 53 patients. One patient did not require fixation because of an old fracture with a stable femoral component. After reaming the femoral canal, a long-stemmed femoral trial prosthesis with a bipolar head was inserted. After joint stability was confirmed, the trial components were removed and the definitive components were implanted (Figure 2).

**Figure 2:**
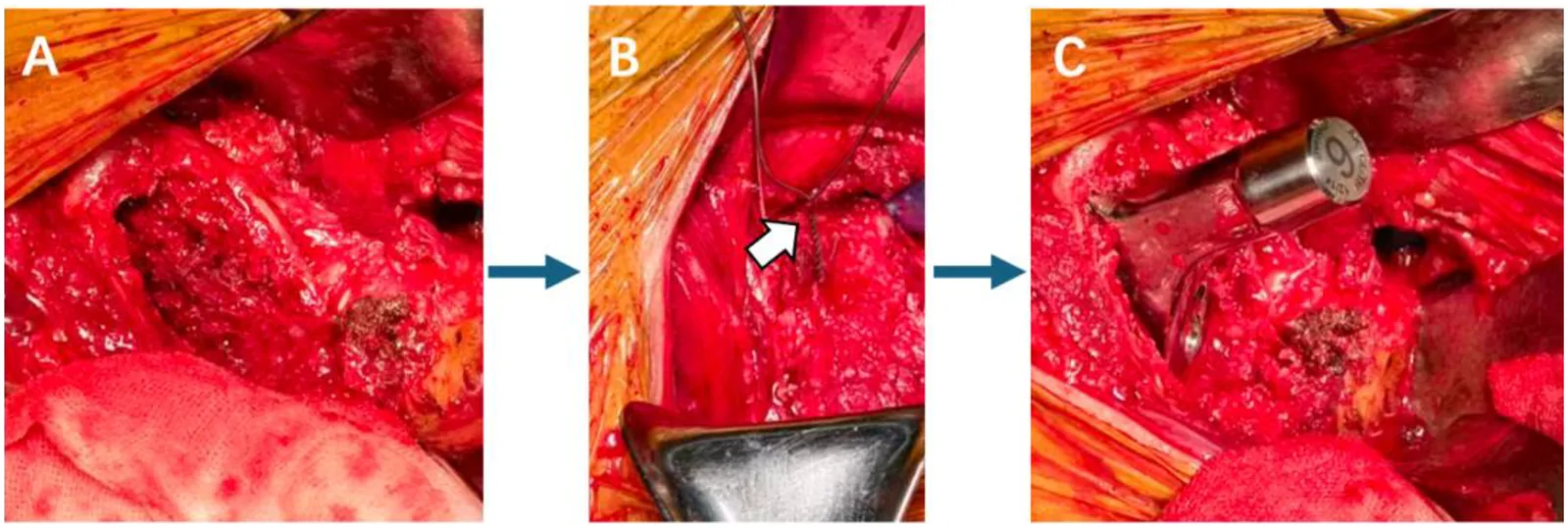
Intraoperative photographs of DAA hemiarthroplasty for trochanteric fractures. **(A)** Exposure of the femoral medullary cavity. **(B)** Fixation of the fractured greater/lesser trochanter with steel wire (white arrow). **(C)** Implantation of the femoral stem.

PFNA internal fixation: The patient was placed on a traction table, and fracture reduction was achieved under C-arm fluoroscopy. Surgery proceeded after satisfactory fracture alignment was confirmed. The apex of the greater trochanter was palpated, and a 4–5 cm incision was made approximately 3 cm proximal to this point. Soft tissues were incised, and muscles were bluntly dissected. A guidewire was inserted at the midpoint of the greater trochanter apex. After fluoroscopic confirmation, an awl was used to create an entry point, followed by sequential reaming of the medullary canal. The PFNA main nail was inserted. A targeting device was attached proximally for lag screw insertion and distally for femoral shaft locking screw insertion.

### 2.4 Clinical evaluation

Postoperative x-ray films were assessed to determine whether loosening was present at the fracture site, to evaluate the healing of the greater trochanter, to assess subsidence, reduction quality, neck-shaft angle, and tip-apex distance (TAD). The fracture reduction in the PFNA group was evaluated by comparing preoperative and postoperative x-rays and the use of Baumgaertner-Fogagnolo classification (17) (Figure 3). The degree of femoral component loosening was assessed according to Engh's method (5, 18). Prosthetic subsidence was indicated by measuring the displacement of the greater trochanter. The displacement distance of the greater trochanter was defined as the difference between the stem-to-tip distance measured on the x-ray image at the last follow-up (Figure 4) (the distance between the highest point of the femoral stem and the highest point of the greater trochanter) and the initially measured stem-to-tip distance (7).

**Figure 3:**
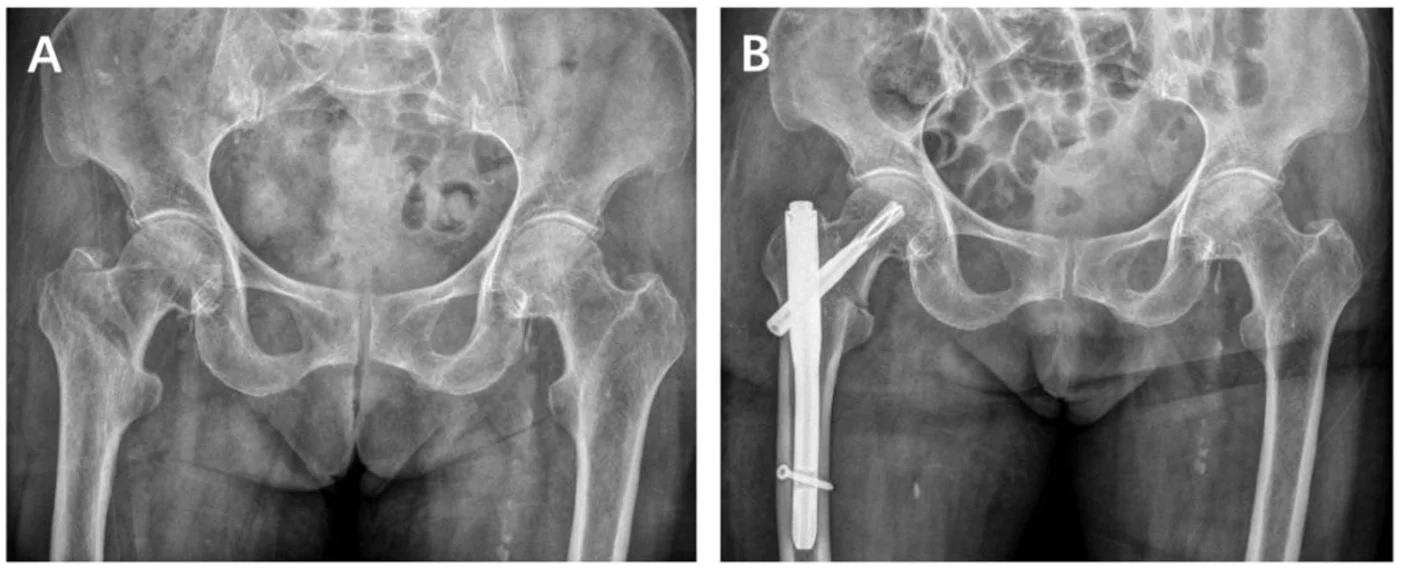
Preoperative **(A)** and postoperative **(B)** radiographs of a patient who underwent PFNA internal fixation.

**Figure 4:**
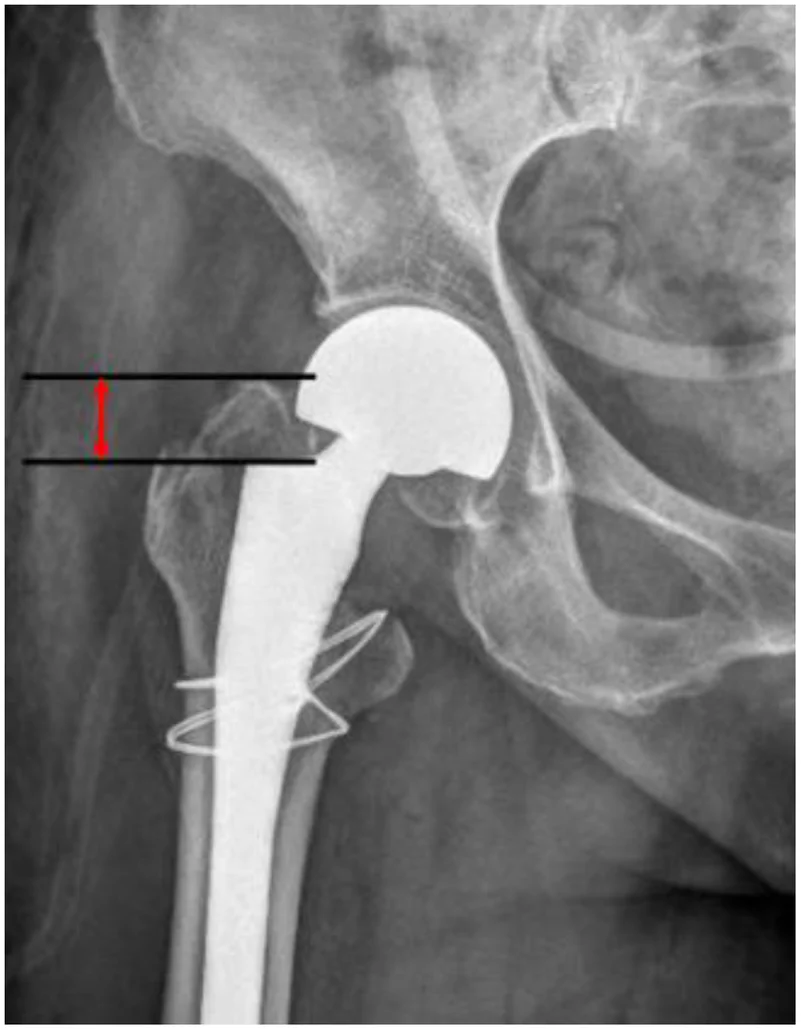
Measurement of femoral component subsidence, defined as the distance between the tip of the femoral stem and the apex of the greater trochanter (red arrow).

All patients received guidance on postoperative rehabilitation exercises from professional rehabilitation physicians. The rehabilitation protocols differed between the two groups. The DAA group underwent standing and walking exercises, as well as muscle strengthening exercises, in the early postoperative period. The PFNA group performed bed-based joint mobility and muscle strengthening exercises, with standing and walking typically initiated 6–8 weeks postoperatively under outpatient guidance. This progressive weight-bearing protocol was designed according to the National Health Commission's Guidelines for the Diagnosis and Management of Geriatric Hip Fractures (2022 edition), which recommends early weight-bearing activity without specifying immediate full weight-bearing, particularly in osteoporotic patients. The HHS was used to assess clinical functional outcomes.

### 2.5 Statistics

Data were analyzed using SPSS 21.0 (SPSS Inc., Chicago, IL, USA). Continuous variables are presented as mean ± SD. To minimize selection bias, 1:1 propensity score matching (PSM) was performed using a logistic regression model based on age, sex, BMI, AO/OTA fracture type, CCI, and ASA grade, with a caliper of 0.2 × SD (logit) and nearest-neighbor matching without replacement. After matching, intergroup comparisons were using independent-samples *t*-tests for continuous variables and chi-square or Fisher's exact tests for categorical variables. A two-sided *P* < 0.05 was considered significant. HHS was compared between groups at each prespecified follow-up time point (1, 3, 6, 12, and 24 months). Between-group mean differences with 95% confidence intervals were calculated in addition to *P* values. A mixed-effects model for repeated measures was not used.

## 3 Results

### 3.1 Preoperative basic data and intraoperative results

After propensity score matching, no statistically significant differences were observed in any baseline characteristics between the two groups (all *P* > 0.05). In the DAA group, the mean age was 81.17 ± 3.92 years with a BMI of 23.31 ± 2.67 kg/m^2^; in the PFNA group, the mean age was 81.58 ± 3.46 years with a BMI of 23.51 ± 2.06 kg/m^2^ (Table 1). Standardized mean differences (SMDs) before and after propensity score matching are shown in Table 2. After matching, all covariates had SMD <0.1, indicating adequate balance.

**Table 2:** Standardized mean differences before and after propensity score matching.

Covariate	SMD before matching	SMD after matching
Age	0.33	0.09
Sex	0.11	0.07
BMI	0.21	0.08
AO/OTA 31-A1	0.14	0.04
AO/OTA 31-A2	0.10	0.04
AO/OTA 31-A3	0.05	0.00
CCI	0.24	0.09
ASA grade	0.20	0.00

SMD, standardized mean difference; BMI, body mass index; AO/OTA, Arbeitsgemeinschaft für Osteosynthesefragen/Orthopaedic Trauma Association; CCI, Charlson Comorbidity Index; ASA, American Society of Anesthesiologists. SMD <0.1 was considered to indicate adequate balance between groups.

Regarding intraoperative outcomes, the DAA group had a mean blood loss of 349.6 ± 124.0 mL, which was significantly higher than that of the PFNA group (220.3 ± 65.2 mL) (*P* < 0.001). The mean operative duration was also longer in the DAA group (75.02 ± 7.19 min vs. 40.05 ± 8.27 min, *P* < 0.001). Conversely, the total incision length was greater in the PFNA group (11.49 ± 0.81 cm vs. 9.29 ± 0.54 cm, *P* < 0.001). Notably, the incision length in the PFNA group represents the sum of 3 to 6 individual incisions (Table 3, Figure 5).

**Table 3:** Intraoperative and early postoperative outcomes.

Result category	DAA group	PFNA group	*P* value
Blood loss (mL)	349.6 ± 124	220.3 ± 65.2	**<0.001**
Duration of surgery (min)	75.02 ± 7.19	40.05 ± 8.27	**<0.001**
Incision length (cm)	9.29 ± 0.54	11.49 ± 0.81	**<0.001**
Bedridden duration (days)	0.99 ± 0.24	52.86 ± 8.95	**<0.001**

Data are shown as mean ± SD.

Bold values indicate statistically significant differences.

**Figure 5:**
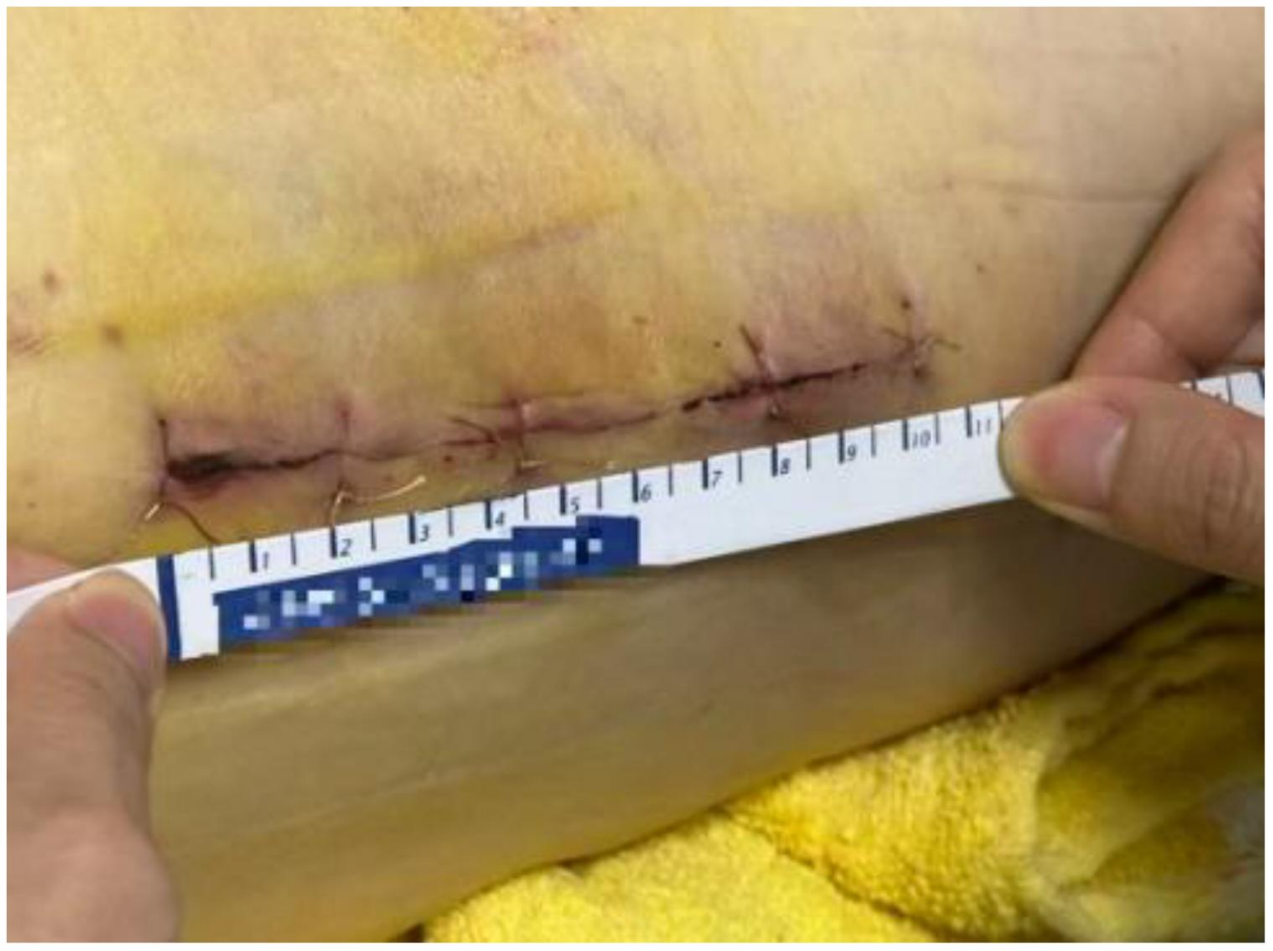
Measurement of the incision length after DAA hemiarthroplasty.

### 3.2 Functional rehabilitation

Functional recovery outcomes are summarized in Table 4 and Figure 6. The mean bedridden duration was significantly shorter in the DAA group than in the PFNA group (0.99 ± 0.24 vs. 52.86 ± 8.95 days, *P* < 0.001).

**Table 4:** Harris Hip Scores (HHS) at postoperative follow-up time points.

Time point	DAA group	PFNA group	Mean difference (95% CI)	*T* value	*P* value	Effect estimate	Post hoc power
1month	78.85 ± 2.67	63.89 ± 2.82	18.96 (13.92 to 15.99)	28.64	**<0.001**	MD 14.96	**>0.99**
3 months	82.39 ± 2.29	71.74 ± 4.35	10.65 (9.33 to 11.97)	16.01	**<0.001**	MD 10.65	**>0.99**
6 months	88.30 ± 2.89	79.75 ± 2.20	8.55 (7.58 to 9.51)	17.58	**<0.001**	MD 8.55	**>0.99**
12 months	91.78 ± 2.06	82.49 ± 2.12	9.29 (8.50 to 10.07)	23.36	**<0.001**	MD 9.29	**>0.99**
24 months	92.03 ± 1.87	82.86 ± 2.16	9.17 (8.42 to 9.93)	24.09	**<0.001**	MD 9.17	**>0.99**

Data are presented as mean ± SD. HHS, Harris hip score; MD, mean difference.

Bold values indicate statistically significant differences.

**Figure 6:**
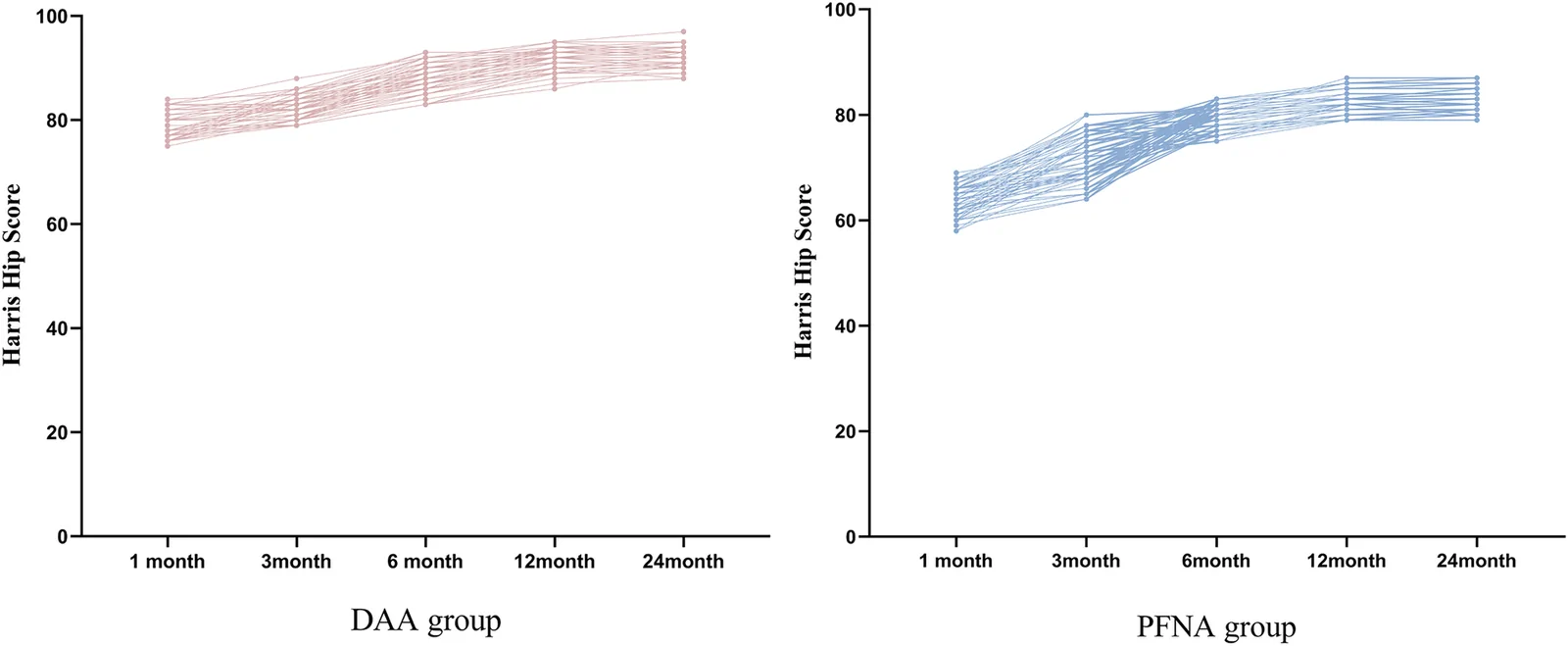
Harris hip scores (HHS) of the two groups at postoperative follow-up time points (1, 3, 6, 12, and 24 months).

The Harris Hip Score (HHS) improved over time in both groups. At each postoperative time point (1, 3, 6, 12, and 24 months), the mean HHS was significantly higher in the DAA group than in the PFNA group (all *P* < 0.001). The mean HHS values for the DAA group were 78.85, 82.39, 88.30, 91.78, and 92.03, respectively; for the PFNA group, the corresponding values were 63.89, 71.74, 79.75, 82.49, and 82.86 (Table 4).

### 3.3 Image results

Imaging findings are presented in Figure 7. At the two-year follow-up, greater trochanter nonunion was observed in 1 patient (1.9%) in the DAA group and 2 patients (3.7%) in the PFNA group, the difference was not statistically significant (*P* = 0.591). In the DAA cohort, the mean prosthesis subsidence was 2.7 mm (range: 0–5.3 mm). No prosthesis loosening was detected on the final follow-up x-ray images in either group. In the PFNA group, the mean TAD was 20.3 mm, and the mean postoperative neck-shaft angle was 129.2° ± 3.0°. Reduction quality was good in 49 patients, acceptable in 5, and poor in 0. Intraoperative fixation of the greater or lesser trochanter was performed in 53 patients (98.1%), while one patient did not require fixation because of an old fracture with a stable femoral component.

**Figure 7:**
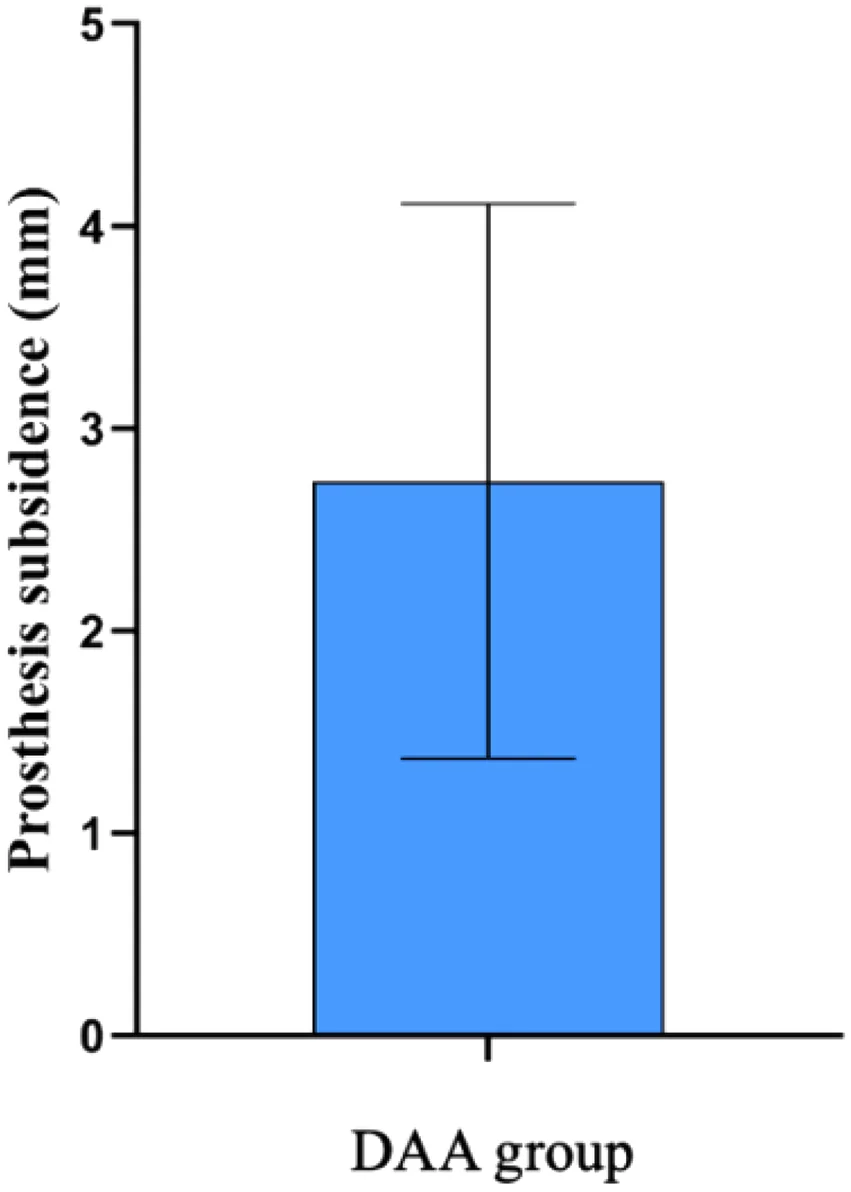
Prosthesis subsidence distribution in the DAA group at the final follow-up.

### 3.4 Complications

Postoperative complications are listed in Table 5. Lower extremity venous thrombosis occurred in 4 patients (7.4%) in the DAA group (all intermuscular venous thrombosis) and in 10 patients (18.5%) in the PFNA group (8 intermuscular, 2 posterior tibial). Pressure sores developed in 1 patient (1.9%) in the DAA group (sacrococcygeal) and in 5 patients (9.3%) in the PFNA group (4 sacrococcygeal, 1 on the healthy hip). Pulmonary complications were documented before discharge in 6 patients (11.1%) in the DAA group and 8 patients (14.8%) in the PFNA group. None of these differences reached statistical significance. There were no perioperative deaths during hospitalization and no deaths during the 2-year follow-up period in either group.

**Table 5:** Postoperative complications.

Complication	DAA group	PFNA group	*P* value
Lower extremity venous thrombosis	4 (7.4%)	10 (18.5%)	0.108
Pressure sores	1 (1.9%)	5 (9.3%)	0.107
Pulmonary complications	6 (11.1%)	8 (14.8%)	0.643
Periprosthetic fractures	0 (0%)	0 (0%)	—
Dislocation	0 (0%)	0 (0%)	—
Loosening of prosthesis	0 (0%)	0 (0%)	—

Data are expressed as *n* (%).

Post hoc power analysis showed high power for the observed HHS differences (>0.99 at both 1 month and 24 months). However, power was limited for complication outcomes: approximately 0.36 for lower extremity venous thrombosis, 0.33 for pressure sores, and 0.08 for pulmonary complications. These findings indicate that the study was underpowered to detect differences in postoperative complications.

## 4 Discussion

The incidence of hip fractures in elderly patients continues to rise, posing challenges for individualized treatment. Most surgeons still prefer internal fixation for Trochanteric fractures (TF) and reserve joint replacement for failed fixation or specific complications (19). However, an increasing number of surgeons consider arthroplasty as a primary option, although evidence comparing different modalities remains insufficient. Previous studies have shown that elderly TF patients can achieve satisfactory outcomes after arthroplasty (20), but they did not control for prosthesis type or surgical approach. Notably, the efficacy of the direct anterior approach (DAA) in TF remains unclear. This study compared DAA hemiarthroplasty with PFNA internal fixation in a propensity score-matched cohort.

Our findings show that the HHS in the PFNA group at 6 months (79.75) was comparable to that in the DAA group at 1 month (78.85), suggesting that DAA hemiarthroplasty may allow earlier functional recovery. However, this finding should be interpreted cautiously because the PFNA group followed a markedly more conservative rehabilitation protocol. Given the realities of clinical practice, it is nearly impossible to administer identical rehabilitation programs for both procedures, making confounding by rehabilitation differences inevitable. This study was designed to compare two distinct comprehensive treatment strategies—DAA hemiarthroplasty vs. PFNA internal fixation—rather than the surgical techniques alone.

The surgical success rate and postoperative outcomes of PFNA internal fixation for femoral TF are widely acknowledged; however, its limitations contribute to low satisfaction among certain patients. Furthermore, relevant studies indicate that the quality of fracture reduction directly influences postoperative patient outcomes, with poor reduction even increasing postoperative mortality (21–23). Joint replacement can circumvent the issues associated with fracture reduction. Joint arthroplasty can be categorized into total hip arthroplasty and hemiarthroplasty. One study demonstrated that utilizing hip arthroplasty for the treatment of TF complicated by osteoporosis yields favorable functional results, with comparable outcomes between total hip and hemiarthroplasty (5). Based on the surgical approach, it can be classified into the posterolateral approach, lateral approach, direct anterior approach, among others. According to our investigation, the majority of previous studies employed the posterolateral and lateral approaches.

We opted to investigate hemiarthroplasty because elderly patients typically have no excessive demands regarding mobility recovery and prosthesis longevity. Moreover, the shorter operation time and reduced blood loss associated with hemiarthroplasty, compared to total hip arthroplasty, represent significant advantages. Different internal fixation methods yield varying postoperative results, with gamma nail (GN) and sliding hip screw (SHS) demonstrating inferior outcomes compared to PFNA (24). PFNA internal fixation is frequently employed in the clinical treatment of TF. Therefore, we excluded other internal fixation methods and selected PFNA for the internal fixation.

In terms of HHS, both groups improved significantly postoperatively. The early postoperative HHS in the DAA group was significantly higher than that in the PFNA group. This difference is largely attributable to the longer bed rest and delayed weight-bearing in the PFNA group. The between-group HHS differences exceeded the commonly cited minimal clinically important difference (MCID) of approximately 8 points for hip arthroplasty. However, because rehabilitation protocols differed substantially, this clinical benefit cannot be attributed to the surgical approach alone. In our institution, the PFNA rehabilitation protocol prescribed bed-based exercises for the first 6–8 weeks, with standing and walking starting only after 6–8 weeks. This progressive weight-bearing regimen was designed according to the National Health Commission's Guidelines for the Diagnosis and Management of Geriatric Hip Fractures (2022 edition), which supports early weight-bearing but does not mandate immediate full weight-bearing, especially in osteoporotic patients (25). The delayed weight-bearing protocol after PFNA was not randomized and may reflect institutional tradition, concerns about osteoporotic bone, or surgeon preference. It is possible that some stable 31-A1 fractures could have been mobilized earlier. In recent years, our institution has adopted more individualized rehabilitation protocols, and most PFNA patients now begin early weight-bearing. Consequently, the PFNA group had a mean bedridden duration of nearly 53 days, which significantly delayed functional recovery compared with the DAA group, where patients were mobilized within one day postoperatively. Kumar et al. defined delayed weight-bearing after PFNA2 surgery as “after 4 weeks” in a prospective randomized controlled study, and found no significant difference in functional recovery at 24 weeks between the delayed weight-bearing group and the early weight-bearing group (26). In practical terms, this study compared DAA hemiarthroplasty followed by immediate mobilization with PFNA followed by delayed weight-bearing. Therefore, the functional outcome gap reflects both the surgical procedure and the rehabilitation strategy. In other words, DAA hemiarthroplasty provides patients with the opportunity for early rehabilitation, which may be an important reason for the differences in clinical outcomes, rather than the surgical differences directly causing the clinical differences. However, this does not contradict the purpose and conclusion of this study, namely that patients who chose DAA achieved faster recovery and better clinical outcomes.

Furthermore, our finding that HHS in the DAA group remained significantly higher even at 2 years (by 9.17 points) contrasts with a previous meta-analysis by Hongku et al., who reported no functional differences beyond 6 months (20). The discrepancy likely arises from differences in surgical approach (DAA vs. mostly posterolateral in previous studies) and the much more aggressive early mobilization protocol in our DAA group. Previous studies have demonstrated that DAA offers faster rehabilitation for femoral neck fractures (14, 15, 27); our study extends this advantage to TF. Nevertheless, long-term outcomes beyond 2 years require further investigation. These differences should be interpreted in the context of both statistical and clinical significance. The HHS difference at 1 month was 14.96 points, and at 24 months was 9.17 points, both exceeding the MCID. Nevertheless, the rehabilitation confounder limits causal attribution.

Our work also documented and compared relevant intraoperative data. PFNA demonstrated significant advantages in terms of operative time and intraoperative blood loss. On average, each PFNA procedure lasted 40.05 min, which was 34.97 min shorter than that of hemiarthroplasty. Additionally, the blood loss per PFNA procedure was approximately 220.3 mL, 129.3 mL less than that of hemiarthroplasty. For patients at higher surgical risk, reducing operative time and blood loss can mitigate risks, particularly in elderly patients who often have multiple underlying conditions. This is one of the reasons why surgeons have traditionally preferred fracture reduction and internal fixation for trochanteric fractures. PFNA may be considered for patients at higher surgical risk but this was not directly tested in the present study and should be regarded as a clinical consideration rather than a study finding. Previous evidence indicates that DAA surgical procedures impose higher demands on surgeons (28). In our cohort, PFNA was associated with shorter operative time and less intraoperative blood loss than DAA hemiarthroplasty. However, surgical difficulty was not objectively measured in this study.

Regarding complications, the incidence rates of prosthesis loosening, periprosthetic fractures, and joint dislocation were all zero. The DAA group exhibited fewer cases of lower limb venous thrombosis, skin pressure sores, and pulmonary complications compared to the PFNA group. However, these differences were not statistically significant in this study, possibly due to the small sample size. Increasing the sample size might reveal more pronounced differences. The sample size of 54 patients per group was relatively small. *Post hoc* power analysis confirmed that the study was adequately powered for the observed HHS differences but underpowered for rare complications. Therefore, the numerically lower complication rates in the DAA group should not be interpreted as evidence that DAA reduces complications. Complication analyses should be regarded as exploratory. Larger multicenter studies with adequate power are needed to compare rare adverse events between DAA hemiarthroplasty and PFNA.

Regarding the imaging results, we primarily documented the healing status of the greater trochanter and the subsidence of the femoral component following joint replacement surgery. In the DAA group, at the final follow-up, the femoral stem exhibited an average subsidence of 2.7 mm compared to the initial postoperative measurement. Migliorini et al. reported an average prosthesis subsidence of 2.8 mm following THA (29), a finding closely aligned with our observations. Bornes et al. posited that prosthesis subsidence is primarily correlated with patients' body mass index (30). In our study, two patients demonstrated prosthesis subsidence exceeding 5 mm, with a maximum of 5.3 mm, which we attribute to body weight and osteoporosis.

Recent studies by Hernández-Pascual et al. have evaluated factors influencing outcomes after cephalomedullary nailing. Their 2025 study found that immediate partial weight bearing postoperatively could negatively affect short-term survival in TF with at least sufficient reduction (31). Their 2021 study showed that distal locking configuration did not influence long-term mobility or survival, though static locking had more minor complications (32). Their 2022 study introduced the GammaTScore for predicting early cut-out and consolidation time (33). These findings underscore the importance of standardized rehabilitation and implant-related factors when interpreting outcomes after PFNA.

In summary, we suggest that for elderly patients with TF, treatment options should be individualized rather than routinely confined to internal fixation. For instance, when patients express a preference for early functional recovery, are unable to tolerate prolonged immobilization, and are medically fit for longer surgical durations, hemiarthroplasty may be considered as a viable alternative, with the DAA approach potentially offering advantages in this context. Fatemeh Ghasemi et al. reported that quality of life following internal fixation of trochanteric fractures was suboptimal in several domains, particularly among elderly patients (34). These findings suggest that internal fixation may not always be the most suitable initial option for all elderly patients with TF, and patient-centered factors should be carefully weighed in surgical decision-making. The present study cannot determine whether the difference in functional recovery was caused by the arthroplasty itself, the DAA approach, the postoperative rehabilitation strategy, or a combination of these factors.

This study has several important limitations. First, despite the use of propensity score matching, the retrospective, non-randomized design is inherently subject to selection bias. Propensity score matching (PSM) can only balance observed covariates. Important unmeasured factors such as pre-injury functional status, frailty, cognitive function, bone mineral density, nutritional status, socioeconomic factors, and surgeon-specific decision-making were not included in the matching model. Second, the most significant confounder is the substantial difference in postoperative rehabilitation protocols between the two groups. The PFNA group followed a progressive weight-bearing regimen (6–8 weeks of bed-based exercises), which is more conservative than the immediate mobilization in the DAA group. This difference, rather than the surgical technique alone, likely contributed substantially to the observed functional outcome gap. Third, the sample size is relatively small, which may have limited statistical power to detect differences in rare complications. Fourth, the follow-up period was only 2 years, which is insufficient to evaluate long-term prosthesis survival or late-onset complications. Fifth, this was a single-center study performed by surgeons with substantial experience in DAA. The results may not be generalizable to centers with less DAA expertise or different learning curves. Sixth, we did not include other surgical approaches (e.g., posterolateral hemiarthroplasty) or total hip arthroplasty, so the comparative effectiveness of DAA vs. other arthroplasty techniques remains unknown. Therefore, our conclusions should be considered hypothesis-generating and require validation in large-scale, prospective, randomized controlled trials.

## 5 Conclusion

In conclusion, in this propensity score-matched retrospective study, DAA hemiarthroplasty combined with early rehabilitation exercise was associated with faster early functional recovery compared with PFNA internal fixation in elderly patients with TF. However, PFNA offered advantages of shorter operative time, less blood loss, and lower surgical complexity. The study was underpowered for rare complications, and complication-related findings should be considered exploratory. Importantly, the two groups differed substantially in postoperative rehabilitation protocols, which may have contributed to the observed functional differences. Given the limitations inherent to the retrospective design and the single-center setting, these findings should be interpreted with caution. Individualized treatment decisions should balance patient preferences, bone quality, general health, and surgeon expertise. Prospective randomized trials with standardized rehabilitation protocols are needed to confirm the potential benefits of DAA hemiarthroplasty for this population.

## Data Availability

The raw data supporting the conclusions of this article will be made available by the authors, without undue reservation.
