# Supplementary material for: Comparison of short- to mid-term outcomes between DAA hemiarthroplasty and PFNA internal fixation in elderly patients with trochanteric fractures: a propensity score-matched retrospective study
Source: Front Surg. 2026 Oct 23;13:1954682. doi: 10.3389/fsurg.2026.1954682 (PMC13739659; doi:10.3389/fsurg.2026.1954682)
Supplement: Supplementary file 1 [file Supplementaryfile1.pdf]

Author's Proof

Before checking your proof, **please read the instructions below**

- Carefully read the entire proof and mark all corrections in the appropriate place, using the Adobe Reader commenting tools (Adobe Help). Do not use the Edit tool, as direct edits could be missed (the PDF was blocked for editing to prevent this); annotate your corrections instead.
- Provide your corrections in a single PDF file or post your comments in the Production Forum, making sure to reference the relevant query/line number. Upload or post all your corrections directly in the Production Forum to avoid any comments being missed.
- We do not accept corrections via email or in the form of edited manuscripts.
- Do not provide scanned or handwritten corrections.
- Before you submit your corrections, make sure that you have checked your proof carefully as once you approve it you won't be able to make any further corrections.
- To ensure timely publication of your article, please submit your corrections within 48 hours. We will inform you if we need anything else; do not contact us to confirm receipt.
- Note that the column alignment at the bottom of each page is not ensured during this Author's Proof stage. The columns will be correctly aligned in the final PDF publication. You may therefore notice small differences in the structure of the Author's Proof PDF versus the final publication.

Do you need help? Visit our Production Help Center for more information. If you can't find an answer to your question, contact your Production team directly by posting in the Production Forum.

NOTE FOR CHINESE-SPEAKING AUTHORS: If you'd like to see a Chinese translation, click on the symbol next to each query. Only respond in English as non-English responses will not be considered. Translated instructions for providing corrections can be found here.

Quick checklist

- ✓☐ **Author names** - Complete, accurate and consistent with your previous publications.
- ✓☐ **Affiliations** - Complete and accurate. Follow this style when applicable: Department, Institute, University, City, Country.
- ✓☐ **Tables** - Make sure the meaning/alignment of your Tables is correct with the applied formatting style.
- ✓☐ **Figures** - Make sure we are using the latest versions.
- ✓☐ **Funding and Acknowledgments** - List all relevant funders and acknowledgments.
- ✓☐ **Conflict of Interest** - Ensure any relevant conflicts are declared.
- ✓☐ **Supplementary files** - Ensure the latest files are published and that no line numbers and tracked changes are visible. Also, the supplementary files should be cited in the article body text.
- ✓☐ **Queries** - You must reply to all **typesetter's queries below** in order for production to proceed.
- ✓☐ **Content** - Read all content carefully and ensure any necessary corrections are made, then **upload them** to the Production Forum.

Author queries form

Query no.	Details required	Author's response
Q1	Confirm that the article title is correct and check that it makes sense. Note that titles/headings are formatted according to Frontiers' style.	Confirmed. The article title is correct.
Q2	There is a discrepancy between the styling of the author names in the submission system and the manuscript. We have used [Yanfeng Jia, Liqun Xu, Hongwei Bao, Jingzhao Hou, Zhao	Correct.

Query no.	Details required	Author's response
	Wang] instead of [Yanfeng Jia, Zhao Wang, Jingzhao Hou, Hongwei Bao, Liqun Xu]. Please confirm that it is correct.	
Q3	The citation and surnames of all authors have been highlighted. Check that they are correct and consistent with your previous publications, and correct them if needed, noting that the format in the author list should be [First name] [Surname]. Please note that this may affect the indexing of your article in repositories such as PubMed.	Confirmed.<br>The author names and surnames are correct and consistent.
Q4	Provide a URL for the LOOP profile for the following authors if they wish this to be linked to the final published version. If they are not yet registered, ensure that they register with Frontiers at the provided link. Liqun Xu, Hongwei Bao, Jingzhao Hou and Zhao Wang. If a URL is not provided, the profile link will not be added to the article. Non-registered authors and authors with profiles set to "Private" will have the default profile image displayed. Note that we will not be able to add profile links after publication.	Please do not ad any profile links
Q5	Confirm that all author affiliations are correctly listed. Per our style guidelines, affiliations are listed sequentially and follow author order. Requests for non-sequential affiliation listing or to add street addresses/postcodes will not be fulfilled. Note that affiliations should reflect those at the time during which the work was undertaken. If adding new affiliations, specify if these should be listed as a present address instead of a regular affiliation. Please note, a present address will be included separately as a footnote and will not be included in the affiliation list.	All author affiliations are correct.
Q6	Ensure you provide an active email address in the correspondence section. Confirm that the email address is correct and free of typos. Any changes to corresponding authors require individual confirmation from all original and added/removed corresponding authors. Please note, Authorship Change Forms are not required for amendments to the correspondence section.	The correspondence email is correct.
Q7	Confirm that the keywords are correct, and keep them to a maximum of eight and a minimum of five. (Note: a keyword can be made up of one or more words.).	The keywords are correct
Q8	Check if the section headers (i.e., section levelling) have been correctly captured.	The section headers are correct.
Q9	Check that all equations and special characters are displayed correctly.	All equations and special characters display correctly.
Q10	Your article has no Supplementary Material; however, there are citations for "Supplementary Table 2". Could you clarify if you have supplementary material to include, and if so, upload it to the forum?	Sorry, there is no "Supplementary Table 2" in this article. The correct content should be "Table 2"

Query no.	Details required	Author's response
Q11	Confirm that the Data Availability statement is accurate. Note that this statement may have been amended to adhere to our Publication Ethics guidelines.	The Data Availability statement is accurate.
Q12	Confirm that the Ethics statement in this proof is correct. If this is not the latest version, please provide a revised Ethics statement. If your article contains human images, it is your responsibility to ensure that it complies with our Policies and Publication Ethics here, including obtaining written informed consent for publication of any potentially identifiable data or images.	The Ethics statement is correct.
Q13	Confirm that the details in the "Author Contributions" section are correct. If any contributions need to be added/edited, choose the appropriate CRedIT roles from the list available here and indicate which one(s) apply. Please be aware that writing roles ("Writing – original draft" and/or "Writing – review & editing") are a requirement for authorship.	The Author Contributions are correct. Writing roles are included.
Q14	Check all grant numbers and funding information in the proof corresponds to your funding application. All funders should be credited, and all grant numbers should be correctly included in this section. Note that if you add any commercial funding, please ensure that the funders involvement/non-involvement in the manuscript is declared. If you provided a positive funding statement but don't provide funding details, then the statement will be updated to say no funding was received.	All grant numbers and funding information are correct.
Q15	Confirm if the text included in the Conflict of Interest statement is correct. Please do not suggest edits to the wording of the final sentence, as this is standard for Frontiers' journal style, per our guidelines: The author(s)/remaining author(s) declared that this work was conducted in the absence of any commercial or financial relationships that could be construed as a potential conflict of interest.	The Conflict of Interest statement is correct.
Q16	Ensure that any supplementary material is correctly published at this link: https://www.frontiersin.org/articles/10.3389/fsurg.2026.1954682/abstract#supplementary-material. Only published supplementary files will be available to download at the link. If the link does not work, you can check the file(s) directly in the Production Forum; the published supplementary files are highlighted in blue with the status "Published". We recommend citing all supplementary files. Please also provide captions for these files, if relevant. If you have any corrections, provide new files and we will publish them in the Forum. Frontiers will deposit ALL supplementary files to FigShare and they will receive a DOI. Notify us of any previously deposited material. If the Supplementary Material files contain identifiable or copyright images, please keep in mind that it is your responsibility, as the author, to ensure you have permission to use the images in the article. Please check this link for information on author responsibilities and the publication images.	The supplementary material link is correct

Query no.	Details required	Author's response
Q17	The references have been renumbered sequentially to conform to Frontiers' requirements. Please check that the renumbering of the citations and references is correct.	Confirmed. The renumbering of citations and references is correct.
Q18	If you decide to use previously published and/or copyrighted figures in your article, please keep in mind that it is your responsibility as the author to obtain the appropriate permissions and licenses to reproduce them, and to follow any citation instructions requested by third-party rights holders. We require attribution for any elements where you are not the original copyright holder. If obtaining the reproduction rights involves the payment of a fee, these charges are to be paid by the authors. Please provide us with the following attribution details in each case: [Citation], [License], and [Source], and confirm whether you have reproduced or adapted the material. Third-party material includes any proprietary text, illustrations, figures, tables, images, artworks, datasets, databases, as well as trademarked logos or brand names, and any material taken from websites, such as: screenshots, photographs, illustrations, maps, icons, clipart, tables, or social media posts*. *Note: Social media posts require permission from the original user/poster before they can be reproduced.	No third-party copyrighted material is used.
Q19	Ensure that all the figures, tables, and captions are correct, and that all figures are of the highest quality/resolution. You may upload improved figures to the Production Forum. If so, please describe in visual terms the exact changes(s) made to help us confirm that the updated version has been used in the finalized proof. Please note that figures and tables must be cited sequentially, per the author guidelines. If your article contains human images, it is your responsibility to ensure that it complies with our Policies and Publication Ethics here, including obtaining written informed consent for publication of any potentially identifiable data or images.	All figures, tables, and captions are correct.
Q20	Table [2] has not been mentioned in the article. Please add the citation ["Table 2"] within the text. Citing numbered content is mandatory. Note that Figures, Tables, Equations, Algorithms, and Boxes must appear in sequence.	The correct position of "Table 2" should be "Supplementary Table 2" in the original text, and the word "Supplementary" should be r emoved.
Q21	Provide a footnote to indicate the meaning of the bold values provided in Tables [3, 4].	Footnote for bold values in Tables 3 and 4: "Bold values indicate statistically significant differences."
Q22	Please confirm that the below mandatory Frontiers AI generated Alt-Text is an accurate visual description of your Figure(s). As part of our open science commitment, Alt-Text will enhance the accessibility of your manuscript. It is a short visual description that allows people using screen reading technology to clearly understand the contents of an image. These Figure Alt-text proposals won't replace your figure captions and will not be visible on your article. If you wish to make any changes, kindly provide the exact revised Alt-Text you would like to use, ensuring that the word-count remains at approximately 100 words for best accessibility results. **Further information on Alt-Text can be found** here.	The Alt-Text is an accurate visual description

Query no.	Details required	Author's response
	**Figure 1 Alt-Text** – Flowchart illustrating the selection process for a study on 1,469 patients with trochanteric fractures, detailing exclusion criteria, treatment group allocation, propensity score matching, final cohort sizes, and statistical analysis endpoints including functional and secondary outcomes, complications, and imaging results.<br>**Figure 2 Alt-Text** – Surgical procedure shown in three panels labeled A, B, and C; panel A depicts an exposed wound, panel B shows surgical tools with a white arrow indicating a specific point, and panel C involves placement of a metallic instrument marked with the number nine.<br>**Figure 3 Alt-Text** – Panel A shows a pelvic X-ray with a left femoral fracture. Panel B shows the same region after surgical intervention, with internal fixation hardware securing the left femur.<br>**Figure 4 Alt-Text** – X-ray showing a hip joint with a metallic hip prosthesis, demonstrating a total hip replacement. Two black horizontal lines with a red double-headed arrow between them indicate a measured distance above the prosthetic femoral head.<br>**Figure 5 Alt-Text** – Surgical incision on human skin with multiple sutures, surrounded by minor bruising, is being measured with a ruler held by two hands, indicating a length of approximately eleven centimeters.<br>**Figure 6 Alt-Text** – Paired line graphs compare Harris Hip Scores from 1 month to 24 months post-operation for DAA and PFNA groups. DAA scores remain higher throughout, while PFNA scores increase more steeply over time.<br>**Figure 7 Alt-Text** – Bar chart showing prosthesis subsidence in millimeters for the DAA group. The blue bar reaches just below 3 millimeters, with error bars extending approximately from 1.5 to 4.1 millimeters.	
Q23	Figure 6 is low quality/distorted. Therefore, please provide higher resolution/corrected images. If your figure is mainly text and lines, you can upload a vector file for the best quality (EPS or PDF).	To enhance clarity, we have reproduced Figure 6. The content is unchanged; only resolution has been improved.

frontiers in Surgery

TYPE Original Research
PUBLISHED xx xx 2026
DOI

Check for updates

OPEN ACCESS

EDITED BY
Luigi Zanna,
Santa Maria Annunziata Hospital, Italy

REVIEWED BY
Gianfilippo Caggiari,
University of Sassari, Italy
Carlos Hernández-Pascual,
University of Salamanca, Spain

*CORRESPONDENCE
Zhao Wang
✉ 13625189389@yzu.edu.cn

†These authors have contributed equally to this work

RECEIVED 31 July 2026
REVISED 06 October 2026
ACCEPTED 09 October 2026
PUBLISHED xx xx 2026

CITATION
Jia Y, Xu L, Bao H, Hou J and Wang Z (2026) Comparison of short- to mid-term outcomes between DAA hemiarthroplasty and PFNA internal fixation in elderly patients with trochanteric fractures: a propensity score-matched retrospective study. Front. Surg. 13:1954682. doi:

COPYRIGHT
© 2026 Jia, Xu, Bao, Hou and Wang. This is an open-access article distributed under the terms of the Creative Commons Attribution License (CC BY). The use, distribution or reproduction in other forums is permitted, provided the original author(s) and the copyright owner(s) are credited and that the original publication in this journal is cited, in accordance with accepted academic practice. No use, distribution or reproduction is permitted which does not comply with these terms.

Comparison of short- to mid-term outcomes between DAA hemiarthroplasty and PFNA internal fixation in elderly patients with trochanteric fractures: a propensity score-matched retrospective study

Yanfeng Jia†, Liqun Xu†, Hongwei Bao, Jingzhao Hou and Zhao Wang*

Department of Orthopaedic Surgery, Jingjiang People's Hospital Affiliated to Yangzhou University, Taizhou, Jiangsu, China

Background: Trochanteric fractures (TF) are common in elderly patients. Although Proximal Femoral Nail Antirotation (PFNA) internal fixation is widely used, hemiarthroplasty via the direct anterior approach (DAA) has attracted increasing interest because of concerns regarding prolonged bed rest and associated complications. This study compares early outcomes of DAA hemiarthroplasty vs. PFNA internal fixation in elderly TF patients.

Methods: Patients aged ≥75 years with TF treated between January 2018 and January 2024 were retrospectively reviewed. After application of the inclusion and exclusion criteria, 54 patients who underwent DAA hemiarthroplasty were identified. To minimize selection bias, 1:1 propensity score matching was performed based on age, sex, body mass index (BMI), fracture type, Charlson Comorbidity Index (CCI), and American Society of Anesthesiologists (ASA) grade, yielding 54 matched patients who received PFNA. Outcomes included operative duration, blood loss, incision length, bedridden duration, Harris Hip Scores (HHS) at 1, 3, 6, 12, and 24 months, complications, and radiographic findings.

Results: After matching, baseline characteristics were well balanced between the two groups. The PFNA group had significantly less blood loss (220.3 vs. 349.6 mL, $P < 0.001$) and a shorter operative time (40.05 vs. 75.02 min, $P < 0.001$), but a longer total incision length (11.49 vs. 9.29 cm, $P < 0.001$). Bedridden duration was markedly shorter in the DAA group (0.99 vs. 52.86 days, $P < 0.001$). At all follow-up points, HHS was significantly higher in the DAA group (1 month: 78.85 vs. 63.89, $P < 0.001$; 24 months: 92.03 vs. 82.86, $P < 0.001$). Complication rates were lower in the DAA group, although the difference did not reach statistical significance. Mean prosthesis subsidence in the DAA group was 2.7 mm.

Conclusion: In this propensity score-matched retrospective study, DAA hemiarthroplasty was associated with faster early functional recovery and a shorter bedridden duration than PFNA internal fixation in elderly patients with TF, but also with a longer operative time and greater blood loss. The substantial difference in postoperative rehabilitation protocols between the two groups may have contributed to the observed gap in functional outcomes.

Study design: Level of evidence, 3.

KEYWORDS

bipolar hemiarthroplasty, direct anterior approach, elderly patients, femoral intertrochanteric fracture, PFNA internal fixation

1 Introduction

Trochanteric fractures (TF) account for about half of all hip fractures, predominantly affecting osteoporotic elderly patients with high mortality (1, 2). The aging population drives a rising TF incidence. TF causes pain, immobility, and inability to stand, severely impairing quality of life. Historically, closed reduction with intramedullary nail fixation—especially Proximal Femoral Nail Antirotation (PFNA)—has been widely used for unstable TF, owing to its minimally invasive nature, good biomechanics, and short operative time (3). However, in osteoporotic elderly patients, PFNA carries elevated risks of fixation failure, screw cutout, varus malunion, and nonunion (4). Even with successful PFNA, prolonged postoperative bed rest worsens prognosis. While not the gold standard, arthroplasty can improve survival and quality of life in selected elderly TF patients (5).

Arthroplasty was traditionally reserved for failed TF fixation, but increasing evidence shows favorable outcomes when used as a primary treatment (6–10). Total hip arthroplasty (THA) offers better durability and may be more suitable for younger or more active patients, whereas hemiarthroplasty is associated with reduces operative time and blood loss, making it preferable for elderly patients without significant acetabular erosion. Accordingly, most arthroplasty patients in our hospital received hemiarthroplasty.

In terms of surgical approach, the posterolateral approach (PLA) has been the most common; however, there has been a notable increase in the number of direct anterior approach (DAA) recently. Previous studies have demonstrated that DAA offers superior early rehabilitation outcomes in the treatment of femoral neck fractures compared to PLA (11–15). These findings suggest that DAA may yield favorable outcomes in treating femoral neck fractures. As an intermuscular surgical pathway, DAA avoids severing the gluteus medius and gluteus minimus muscles, offering potential advantages such as mild postoperative pain, a low dislocation rate, and rapid gait recovery. Nevertheless, few studies have documented the efficacy of DAA arthroplasty in the management of TF. Theoretically, combining the DAA with hemiarthroplasty can offer older patients more stable fixation and earlier functional recovery. Despite the respective strengths and weaknesses of these two surgical modalities, current comparative studies on the efficacy of DAA hemiarthroplasty vs. PFNA internal fixation for TF in the elderly remain relatively scarce.

This study aims to provide a reference for clinical procedure selection by retrospectively comparing and analyzing the clinical efficacy of DAA hemiarthroplasty and PFNA internal fixation in the treatment of TF in elderly patients. Despite these technical challenges, our team has accumulated extensive experience with the DAA for femoral neck fractures. We hypothesized that its muscle-sparing nature could potentially offer accelerated rehabilitation benefits even in the more complex setting of TF.

2 Material and methods

2.1 Patients

This retrospective study was approved by the Ethics Committee of Jingjiang People's Hospital Affiliated to Yangzhou University. From January 2018 to January 2024, a total of 1,469 consecutive patients with Trochanteric fractures (TF) were treated at our institution. Among them, 1,237 underwent PFNA internal fixation, 44 underwent total hip arthroplasty, and 96 underwent hemiarthroplasty (61 via direct anterior approach [DAA] and 35 via posterolateral approach). The remaining patients received conservative treatment or other internal fixation methods. The inclusion criteria were: (1) unilateral TF; (2) age ≥75 years; (3) treatment with either DAA hemiarthroplasty or PFNA internal fixation. The exclusion criteria were: (1) inability to live independently before injury; (2) pre-existing hip joint disease (e.g., osteoarthritis, rheumatoid arthritis, femoral head necrosis). Of the 61 patients treated with DAA hemiarthroplasty, 7 were excluded before matching: non-compliance with inclusion criteria ($n = 3$), meeting exclusion criteria ($n = 2$), loss to follow-up ($n = 1$), incomplete or missing data ($n = 1$). The remaining 54 patients with complete follow-up data were included. Missing follow-up data were handled by exclusion before propensity score matching. From the initial pool of 1,237 patients treated with PFNA, a total of 892 patients met the same inclusion and exclusion criteria (Figure 1).

To minimize selection bias, 1:1 propensity score matching (PSM) was performed using a logistic regression model. The propensity score for each patient was calculated based on the

---

Abbreviations
DAA, Direct anterior approach; PFNA, Proximal Femoral Nail Antirotation; HHS, Harris Hip Score; TF, Trochanteric fracture; THA, Total Hip Arthroplasty; PSM, Propensity Score Matching; ASA, American Society of Anesthesiologists Physical Status Classification; CCI, Charlson Comorbidity Index; AO/OTA, Arbeitsgemeinschaft für Osteosynthesefragen/Orthopaedic Trauma Association; BMI, Body Mass Index; SD, Standard Deviation; MCID, Minimal Clinically Important Difference.

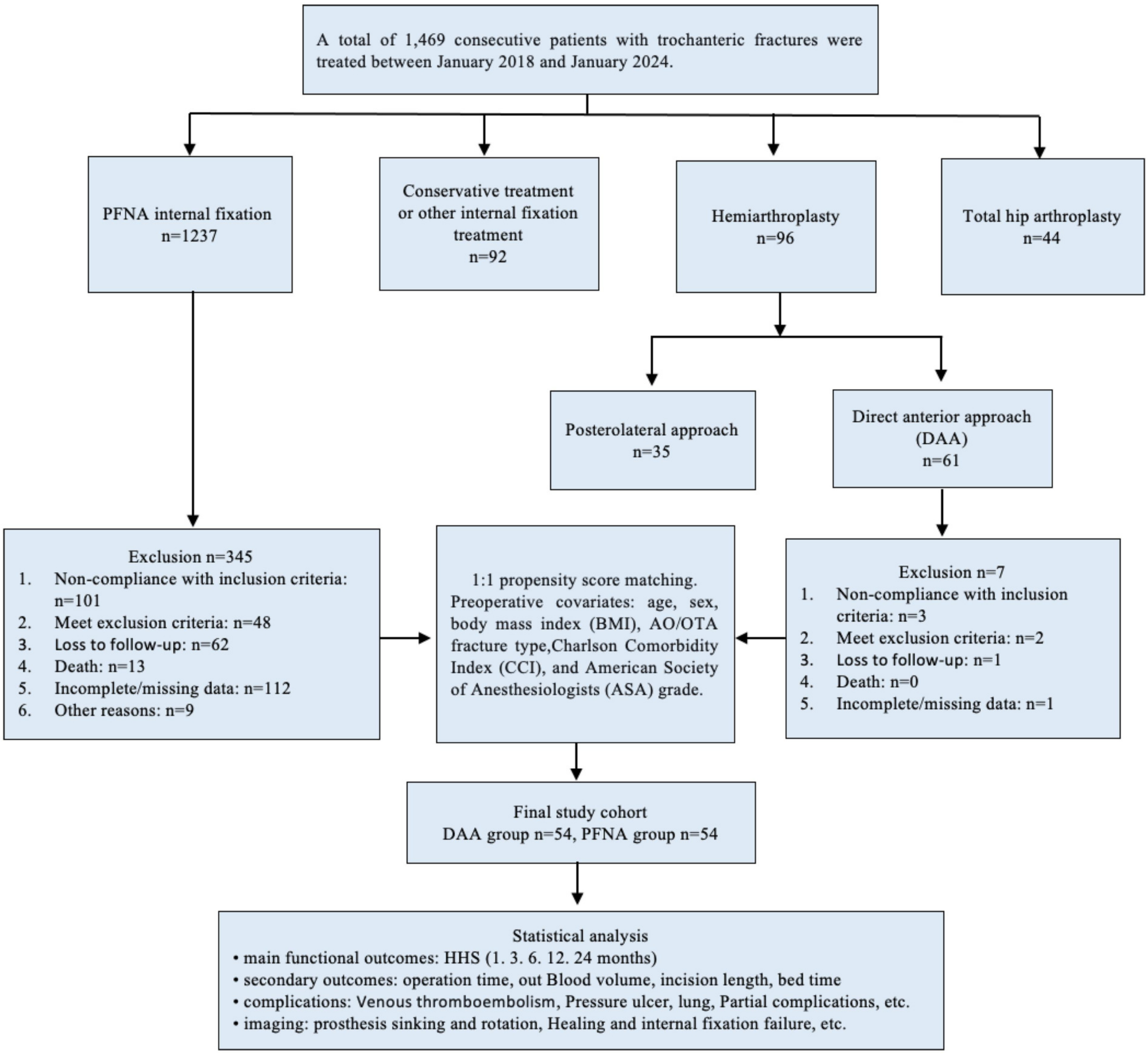

FIGURE 1
Patient flow diagram.

following preoperative covariates: age, sex, body mass index (BMI), fracture type classified according to the AO/OTA system (all included fractures were AO/OTA 31-A fractures), Charlson Comorbidity Index (CCI), and American Society of Anesthesiologists (ASA) grade (16). Unmeasured confounders may persist. Nearest-neighbor matching was applied with a caliper width of 0.2 times the standard deviation of the logit of the propensity score. This process matched 54 patients treated with PFNA to 54 patients treated with DAA, yielding a final study cohort of 108 patients (54 per group). Treatment selection was not randomized. The choice between DAA hemiarthroplasty and PFNA was made by the treating surgeon in consultation with the patient and family and was influenced by medical comorbidity, fracture pattern, bone quality, pre-injury mobility, expected tolerance of bed rest, and patient preference. This represents a potential source of selection bias.

The sample size was determined by the number of eligible patients after propensity score matching rather than by an priori power calculation. *Post hoc* power analysis was performed using the observed matched sample size (54 patients per group) and the observed effect estimates.

Baseline characteristics after matching were well balanced between the two groups (Table 1). In the DAA group, a long-stem femoral component was used in all patients (51 cementless stems and 3 cemented stems). A bipolar head was used in all patients. Intraoperative fixation of the greater or lesser trochanter was performed in 53 patients, while one patient did not require fixation due to an old fracture with a stable femoral component. In the PFNA group, closed reduction was achieved in 53 of the 54 matched patients, with only one case requiring open reduction.

2.2 Data collection

This study documented the intraoperative findings and the 2-year postoperative follow-up results for all subjects. Data

TABLE 1 Baseline demographic and clinical characteristics of the matched cohort.

Characteristic	DAA group	PFNA group	*P* value
Number of patients (*n*)	54	54	—
Sex (female/male)	31/23	33/21	—
Age	81.17 ± 3.92	81.58 ± 3.46	0.558
BMI (kg/m^2)	23.31 ± 2.67	23.51 ± 2.06	0.659
AO/OTA fracture type, *n* (%)			0.721
31-A1 (stable)	18 (33.3%)	17 (31.5%)	
31-A2 (unstable)	28 (51.9%)	29 (53.7%)	
31-A3 (reverse oblique)	8 (14.8%)	8 (14.8%)	
CCI	2.1 ± 1.2	2.0 ± 1.1	0.654
ASA grade, *n* (%)			0.592
II	15 (27.8%)	16 (29.6%)	
III	34 (63.0%)	33 (61.1%)	
IV	5 (9.3%)	5 (9.3%)	

SD, standard deviation; BMI, body mass index; AO/OTA, Arbeitsgemeinschaft für Osteosynthesefragen/Orthopaedic Trauma Association; ASA, American Society of Anesthesiologists; CCI, Charlson Comorbidity Index.

Q20 TABLE 2 Standardized mean differences before and after propensity score matching.

Covariate	SMD before matching	SMD after matching
Age	0.33	0.09
Sex	0.11	0.07
BMI	0.21	0.08
AO/OTA 31-A1	0.14	0.04
AO/OTA 31-A2	0.10	0.04
AO/OTA 31-A3	0.05	0.00
CCI	0.24	0.09
ASA grade	0.20	0.00

SMD, standardized mean difference; BMI, body mass index; AO/OTA, Arbeitsgemeinschaft für Osteosynthesefragen/Orthopaedic Trauma Association; CCI, Charlson Comorbidity Index; ASA, American Society of Anesthesiologists. SMD <0.1 was considered to indicate adequate balance between groups.

collection was conducted collaboratively by two authors. Intraoperative data encompassed the duration of the operation, blood loss volume, and incision length. Postoperative data were categorized into functional outcomes and imaging findings. Functional outcomes included postoperative bed rest duration, Harris Hip Scores (HHS), complications, and rehabilitation outcomes, whereas imaging findings were obtained from measurements on x-ray images. Relevant data were documented at 1 month, 3 months, 6 months, 12 months, and 24 months postoperatively. HHS and radiographic assessments were performed by independent assessors blinded to treatment group. Perioperative mortality during hospitalization and mortality during the 2-year follow-up period were recorded. Pulmonary complications were defined as pneumonia, respiratory failure, acute exacerbation of chronic obstructive pulmonary disease, or pleural effusion. Venous thrombosis was diagnosed by Doppler ultrasonography, which was performed systematically in all patients before discharge. All patients received low-molecular-weight heparin for 7 days after surgery, combined with early mobilization and intermittent pneumatic compression.

2.3 Surgical techniques

Both surgical procedures were performed by four experienced surgeons at our hospital.

DAA hemiarthroplasty: The patient was positioned supine. An approximately 8–10 cm incision was made proximally along the line connecting the anterior superior iliac spine and the lateral femoral condyle. After incising the skin and subcutaneous tissue, the fascia of the tensor fasciae latae was cut and reflected medially toward the space of the rectus femoris. The branch of the lateral femoral circumflex artery was ligated. A specifically designed acetabular retractor was used to expose the anterolateral joint capsule, which was then incised for osteotomy. The femoral head was excised, and the capsule was released. The operating table was flexed to position the lower limb in hyperextension, adduction, and external rotation, thereby adequately exposing the proximal femur. The greater or lesser trochanter was reconstructed and fixed with steel wires or titanium cables in 53 patients. One patient did not require fixation because of an old fracture with a stable femoral component. After reaming the femoral canal, a long-stemmed femoral trial prosthesis with a bipolar head was inserted. After joint stability was confirmed, the trial components were removed and the definitive components were implanted (Figure 2).

PFNA internal fixation: The patient was placed on a traction table, and fracture reduction was achieved under C-arm fluoroscopy. Surgery proceeded after satisfactory fracture alignment was confirmed. The apex of the greater trochanter was palpated, and a 4–5 cm incision was made approximately 3 cm proximal to this point. Soft tissues were incised, and muscles were bluntly dissected. A guidewire was inserted at the midpoint of the greater trochanter apex. After fluoroscopic confirmation, an awl was used to create an entry point, followed by sequential reaming of the medullary canal. The PFNA main nail was inserted. A targeting device was attached proximally for lag screw insertion and distally for femoral shaft locking screw insertion.

2.4 Clinical evaluation

Postoperative x-ray films were assessed to determine whether loosening was present at the fracture site, to evaluate the healing of the greater trochanter, to assess subsidence, reduction quality, neck-shaft angle, and tip-apex distance (TAD). The fracture reduction in the PFNA group was evaluated by comparing preoperative and postoperative x-rays and the use of Baumgaertner-Fogagnolo classification (17) (Figure 3). The degree of femoral component loosening was assessed according

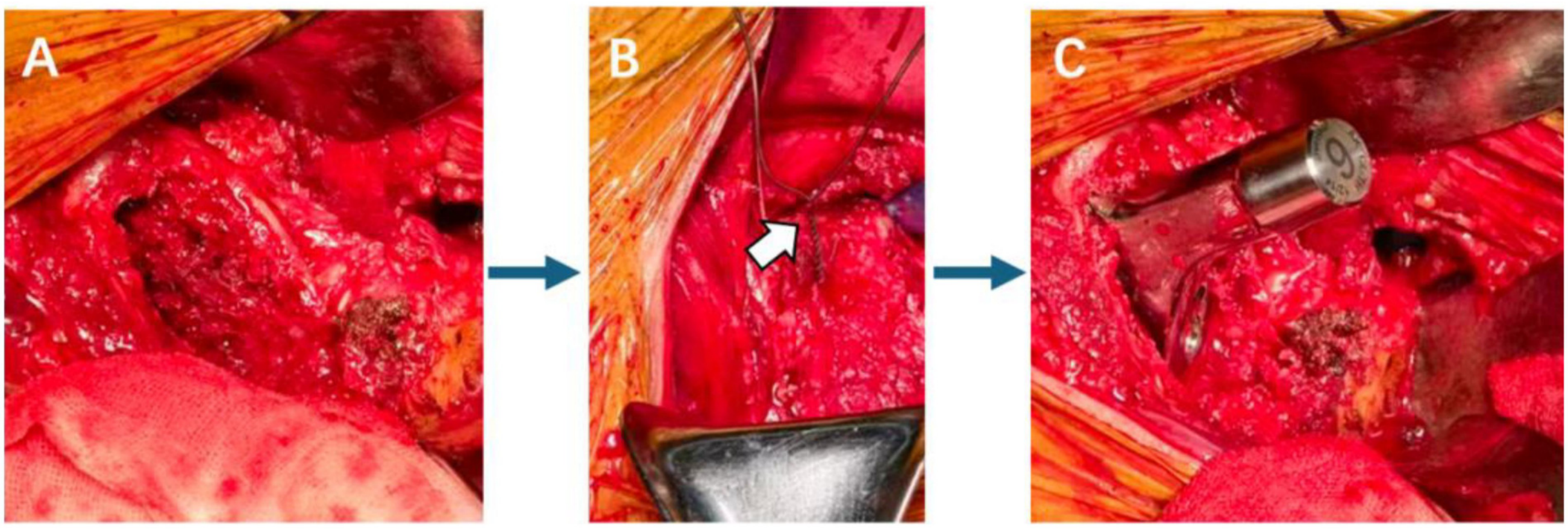

FIGURE 2
Intraoperative photographs of DAA hemiarthroplasty for trochanteric fractures. **(A)** Exposure of the femoral medullary cavity. **(B)** Fixation of the fractured greater/lesser trochanter with steel wire (white arrow). **(C)** Implantation of the femoral stem.

to Engh's method (5, 18). Prosthetic subsidence was indicated by measuring the displacement of the greater trochanter. The displacement distance of the greater trochanter was defined as the difference between the stem-to-tip distance measured on the x-ray image at the last follow-up (Figure 4) (the distance between the highest point of the femoral stem and the highest point of the greater trochanter) and the initially measured stem-to-tip distance (7).

All patients received guidance on postoperative rehabilitation exercises from professional rehabilitation physicians. The rehabilitation protocols differed between the two groups. The DAA group underwent standing and walking exercises, as well as muscle strengthening exercises, in the early postoperative period. The PFNA group performed bed-based joint mobility and muscle strengthening exercises, with standing and walking typically initiated 6–8 weeks postoperatively under outpatient guidance. This progressive weight-bearing protocol was designed according to the National Health Commission's Guidelines for the Diagnosis and Management of Geriatric Hip Fractures (2022 edition), which recommends early weight-bearing activity without specifying immediate full weight-bearing, particularly in osteoporotic patients. The HHS was used to assess clinical functional outcomes.

2.5 Statistics

Data were analyzed using SPSS 21.0 (SPSS Inc., Chicago, IL, USA). Continuous variables are presented as mean ± SD. To minimize selection bias, 1:1 propensity score matching (PSM) was performed using a logistic regression model based on age, sex, BMI, AO/OTA fracture type, CCI, and ASA grade, with a caliper of 0.2 × SD (logit) and nearest-neighbor matching without replacement. After matching, intergroup comparisons were using independent-samples *t*-tests for continuous variables and chi-square or Fisher's exact tests for categorical variables. A two-sided $P < 0.05$ was considered significant. HHS was compared between groups at each prespecified follow-up time point (1, 3, 6, 12, and 24 months). Between-group mean differences with 95% confidence intervals were calculated in addition to *P* values. A mixed-effects model for repeated measures was not used.

3 Results

3.1 Preoperative basic data and intraoperative results

After propensity score matching, no statistically significant differences were observed in any baseline characteristics between the two groups (all $P > 0.05$). In the DAA group, the mean age was 81.17 ± 3.92 years with a BMI of 23.31 ± 2.67 kg/m^2; in the PFNA group, the mean age was 81.58 ± 3.46 years with a BMI of 23.51 ± 2.06 kg/m^2 (Table 1). Standardized mean differences (SMDs) before and after propensity score matching are shown in ~~Supplementary~~ Table 2. After matching, all covariates had Q10 SMD <0.1, indicating adequate balance.

Regarding intraoperative outcomes, the DAA group had a mean blood loss of 349.6 ± 124.0 mL, which was significantly higher than that of the PFNA group (220.3 ± 65.2 mL) ($P < 0.001$). The mean operative duration was also longer in the DAA group (75.02 ± 7.19 min vs. 40.05 ± 8.27 min, $P < 0.001$). Conversely, the total incision length was greater in the PFNA group (11.49 ± 0.81 cm vs. 9.29 ± 0.54 cm, $P < 0.001$). Notably, the incision length in the PFNA group represents the sum of 3 to 6 individual incisions (Table 3, Figure 5).

3.2 Functional rehabilitation

Functional recovery outcomes are summarized in Table 4 and Figure 6. The mean bedridden duration was significantly shorter in the DAA group than in the PFNA group (0.99 ± 0.24 vs. 52.86 ± 8.95 days, $P < 0.001$).

The Harris Hip Score (HHS) improved over time in both groups. At each postoperative time point (1, 3, 6, 12, and 24 months), the mean HHS was significantly higher in the DAA group than in the PFNA group (all $P < 0.001$). The mean HHS values for the DAA group were 78.85, 82.39, 88.30, 91.78, and

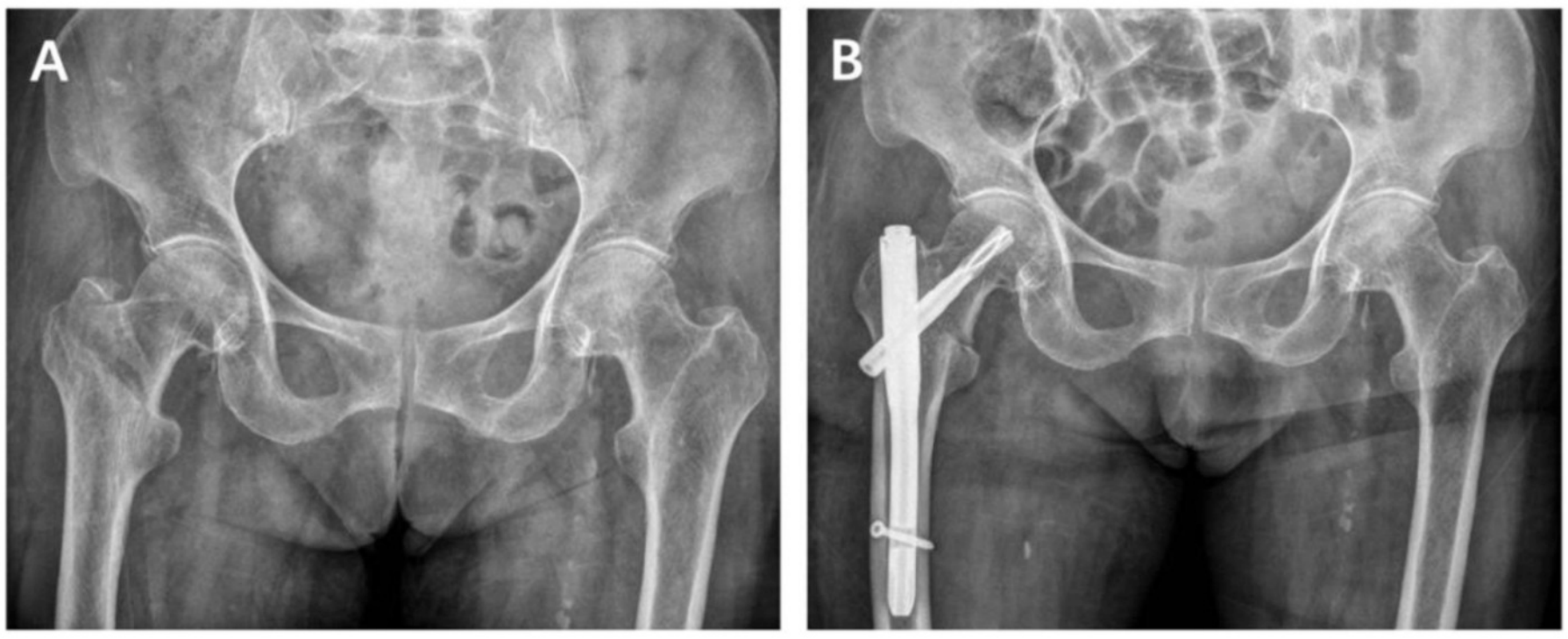

FIGURE 3
Preoperative **(A)** and postoperative **(B)** radiographs of a patient who underwent PFNA internal fixation.

92.03, respectively; for the PFNA group, the corresponding values were 63.89, 71.74, 79.75, 82.49, and 82.86 (Table 4).

3.3 Image results

Imaging findings are presented in Figure 7. At the two-year follow-up, greater trochanter nonunion was observed in 1 patient (1.9%) in the DAA group and 2 patients (3.7%) in the PFNA group, the difference was not statistically significant ($P = 0.591$). In the DAA cohort, the mean prosthesis subsidence was 2.7 mm (range: 0–5.3 mm). No prosthesis loosening was detected on the final follow-up x-ray images in either group. In the PFNA group, the mean TAD was 20.3 mm, and the mean postoperative neck-shaft angle was 129.2° ± 3.0°. Reduction quality was good in 49 patients, acceptable in 5, and poor in 0. Intraoperative fixation of the greater or lesser trochanter was performed in 53 patients (98.1%), while one patient did not require fixation because of an old fracture with a stable femoral component.

3.4 Complications

Postoperative complications are listed in Table 5. Lower extremity venous thrombosis occurred in 4 patients (7.4%) in the DAA group (all intermuscular venous thrombosis) and in 10 patients (18.5%) in the PFNA group (8 intermuscular, 2 posterior tibial). Pressure sores developed in 1 patient (1.9%) in the DAA group (sacrococcygeal) and in 5 patients (9.3%) in the PFNA group (4 sacrococcygeal, 1 on the healthy hip). Pulmonary complications were documented before discharge in 6 patients (11.1%) in the DAA group and 8 patients (14.8%) in the PFNA group. None of these differences reached statistical significance. There were no perioperative deaths during hospitalization and no deaths during the 2-year follow-up period in either group.

Post hoc power analysis showed high power for the observed HHS differences (>0.99 at both 1 month and 24 months). However, power was limited for complication outcomes: approximately 0.36 for lower extremity venous thrombosis, 0.33 for pressure sores, and 0.08 for pulmonary complications. These findings indicate that the study was underpowered to detect differences in postoperative complications.

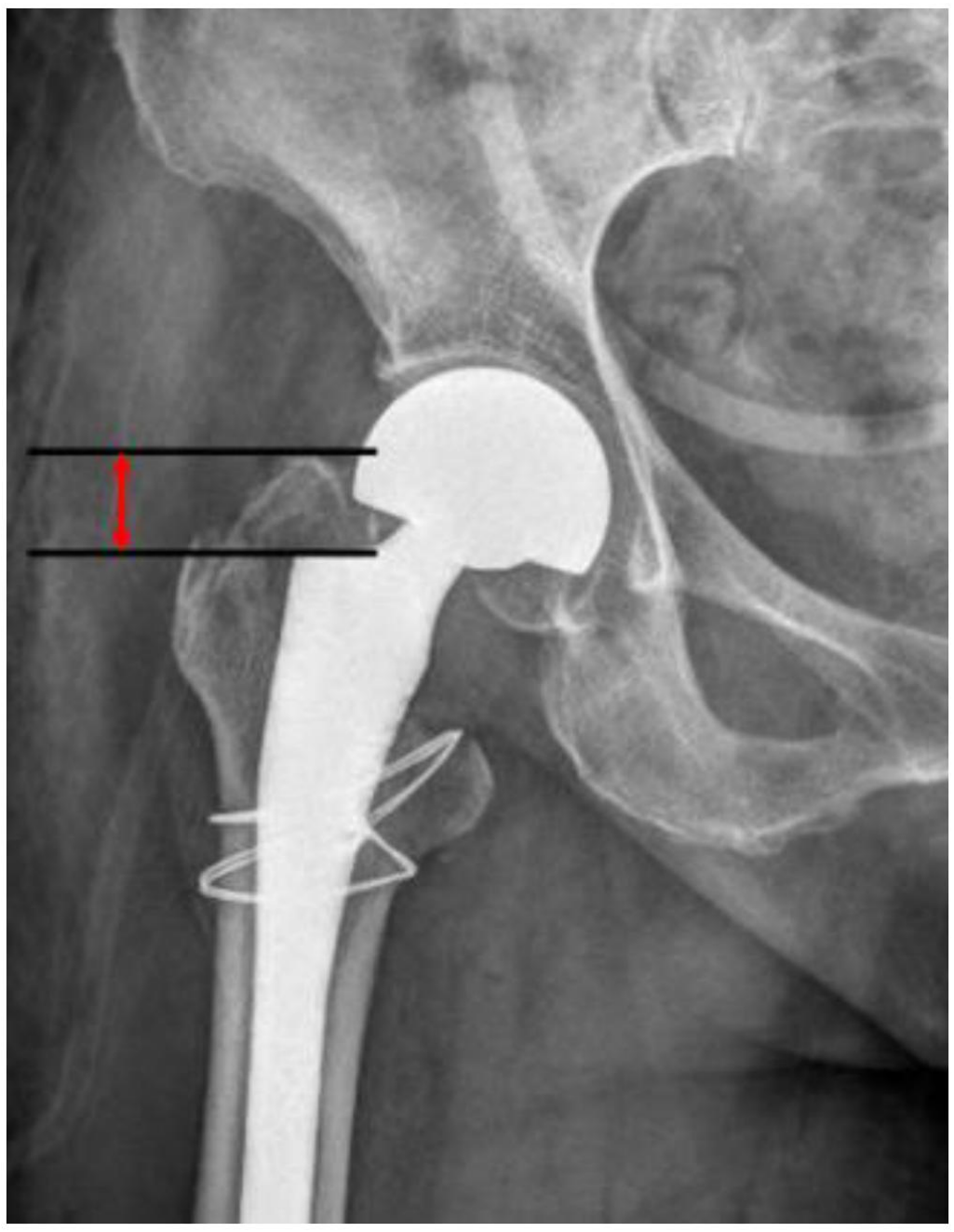

FIGURE 4
Measurement of femoral component subsidence, defined as the distance between the tip of the femoral stem and the apex of the greater trochanter (red arrow).

TABLE 3 Intraoperative and early postoperative outcomes. Q21

Result category	DAA group	PFNA group	*P* value
Blood loss (mL)	349.6 ± 124	220.3 ± 65.2	**<0.001**
Duration of surgery (min)	75.02 ± 7.19	40.05 ± 8.27	**<0.001**
Incision length (cm)	9.29 ± 0.54	11.49 ± 0.81	**<0.001**
Bedridden duration (days)	0.99 ± 0.24	52.86 ± 8.95	**<0.001**

Data are shown as mean ± SD.

4 Discussion

The incidence of hip fractures in elderly patients continues to rise, posing challenges for individualized treatment. Most

surgeons still prefer internal fixation for Trochanteric fractures (TF) and reserve joint replacement for failed fixation or specific complications (19). However, an increasing number of surgeons consider arthroplasty as a primary option, although evidence comparing different modalities remains insufficient. Previous studies have shown that elderly TF patients can achieve satisfactory outcomes after arthroplasty (20), but they did not control for prosthesis type or surgical approach. Notably, the efficacy of the direct anterior approach (DAA) in TF remains unclear. This study compared DAA hemiarthroplasty with PFNA internal fixation in a propensity score-matched cohort.

Our findings show that the HHS in the PFNA group at 6 months (79.75) was comparable to that in the DAA group at 1 month (78.85), suggesting that DAA hemiarthroplasty may allow earlier functional recovery. However, this finding should be interpreted cautiously because the PFNA group followed a markedly more conservative rehabilitation protocol. Given the realities of clinical practice, it is nearly impossible to administer identical rehabilitation programs for both procedures, making confounding by rehabilitation differences inevitable. This study was designed to compare two distinct comprehensive treatment strategies—DAA hemiarthroplasty vs. PFNA internal fixation—rather than the surgical techniques alone.

The surgical success rate and postoperative outcomes of PFNA internal fixation for femoral TF are widely acknowledged; however, its limitations contribute to low satisfaction among certain patients. Furthermore, relevant studies indicate that the quality of fracture reduction directly influences postoperative patient outcomes, with poor reduction even increasing

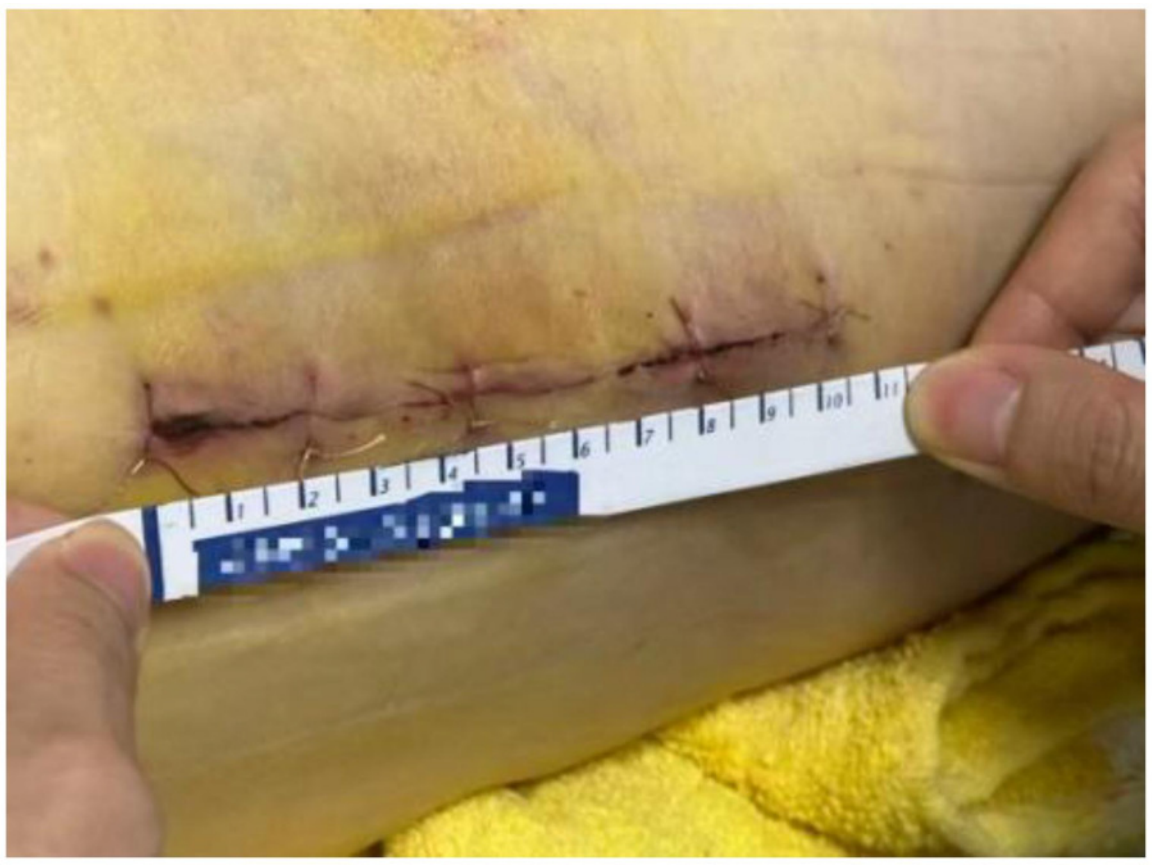

FIGURE 5
Measurement of the incision length after DAA hemiarthroplasty.

TABLE 4 Harris Hip Scores (HHS) at postoperative follow-up time points.

Time point	DAA group	PFNA group	Mean difference (95% CI)	*T* value	*P* value	Effect estimate	Post hoc power
1month	78.85 ± 2.67	63.89 ± 2.82	18.96 (13.92 to 15.99)	28.64	**<0.001**	MD 14.96	**>0.99**
3 months	82.39 ± 2.29	71.74 ± 4.35	10.65 (9.33 to 11.97)	16.01	**<0.001**	MD 10.65	**>0.99**
6 months	88.30 ± 2.89	79.75 ± 2.20	8.55 (7.58 to 9.51)	17.58	**<0.001**	MD 8.55	**>0.99**
12 months	91.78 ± 2.06	82.49 ± 2.12	9.29 (8.50 to 10.07)	23.36	**<0.001**	MD 9.29	**>0.99**
24 months	92.03 ± 1.87	82.86 ± 2.16	9.17 (8.42 to 9.93)	24.09	**<0.001**	MD 9.17	**>0.99**

Data are presented as mean ± SD. HHS, Harris hip score; MD, mean difference.

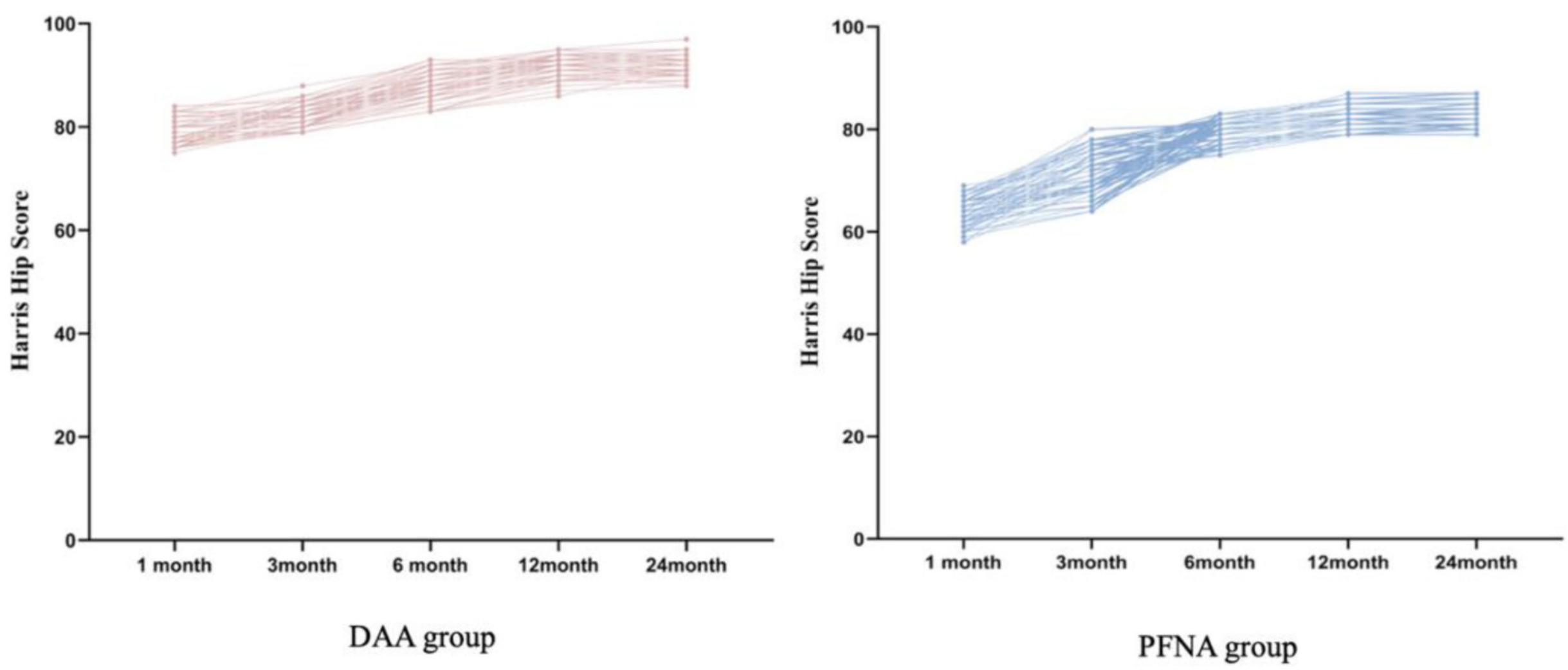

FIGURE 6
Q23 Harris hip scores (HHS) of the two groups at postoperative follow-up time points (1, 3, 6, 12, and 24 months).

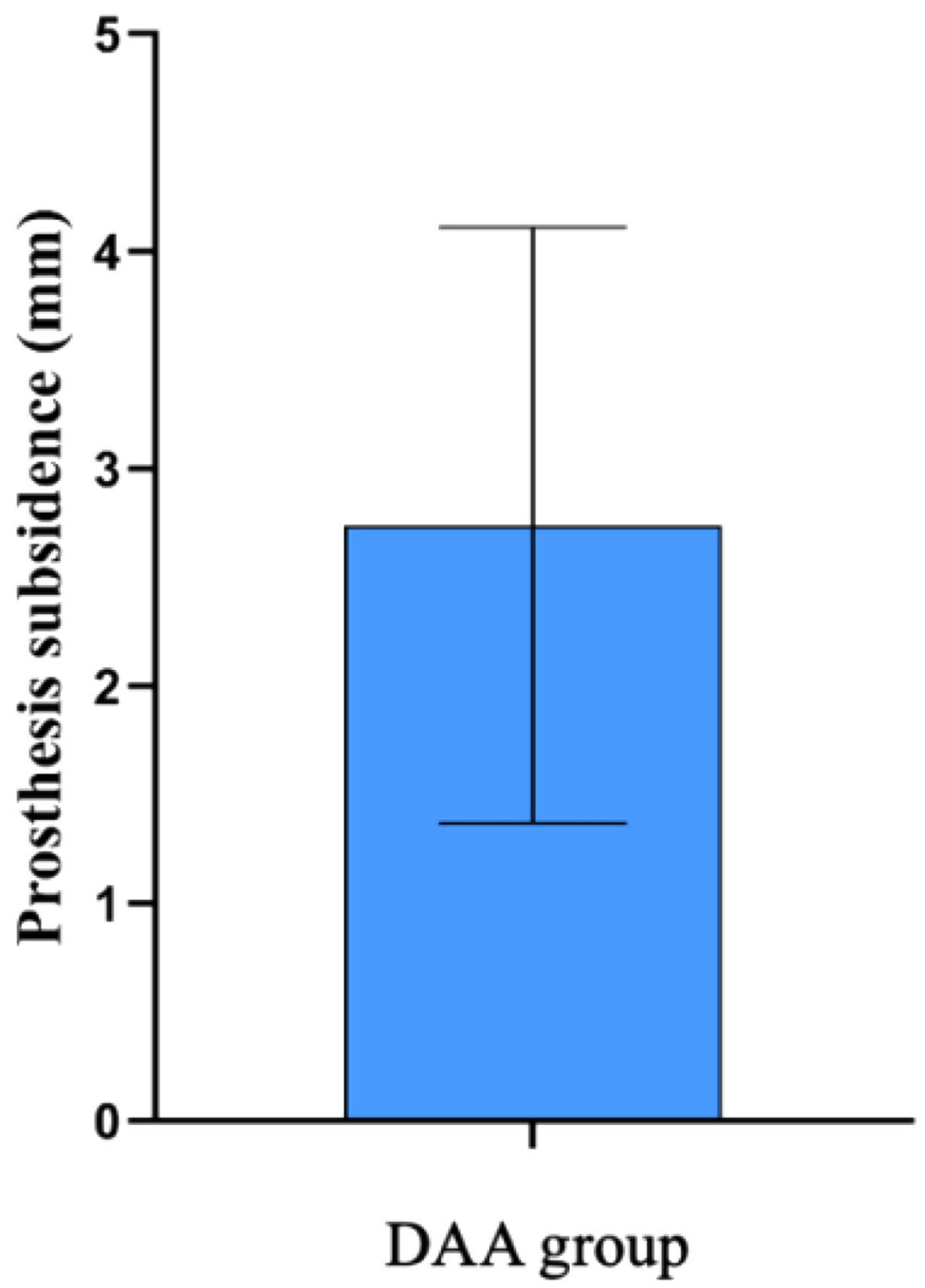

FIGURE 7
Prosthesis subsidence distribution in the DAA group at the final follow-up.

TABLE 5 Postoperative complications.

Complication	DAA group	PFNA group	*P* value
Lower extremity venous thrombosis	4 (7.4%)	10 (18.5%)	0.108
Pressure sores	1 (1.9%)	5 (9.3%)	0.107
Pulmonary complications	6 (11.1%)	8 (14.8%)	0.643
Periprosthetic fractures	0 (0%)	0 (0%)	—
Dislocation	0 (0%)	0 (0%)	—
Loosening of prosthesis	0 (0%)	0 (0%)	—

Data are expressed as *n* (%).

postoperative mortality (21–23). Joint replacement can circumvent the issues associated with fracture reduction. Joint arthroplasty can be categorized into total hip arthroplasty and hemiarthroplasty. One study demonstrated that utilizing hip arthroplasty for the treatment of TF complicated by osteoporosis yields favorable functional results, with comparable outcomes between total hip and hemiarthroplasty (5). Based on the surgical approach, it can be classified into the posterolateral approach, lateral approach, direct anterior approach, among others. According to our investigation, the majority of previous studies employed the posterolateral and lateral approaches.

We opted to investigate hemiarthroplasty because elderly patients typically have no excessive demands regarding mobility recovery and prosthesis longevity. Moreover, the shorter operation time and reduced blood loss associated with hemiarthroplasty, compared to total hip arthroplasty, represent significant advantages. Different internal fixation methods yield varying postoperative results, with gamma nail (GN) and sliding hip screw (SHS) demonstrating inferior outcomes compared to PFNA (24). PFNA internal fixation is frequently employed in the clinical treatment of TF. Therefore, we excluded other internal fixation methods and selected PFNA for the internal fixation.

In terms of HHS, both groups improved significantly postoperatively. The early postoperative HHS in the DAA group was significantly higher than that in the PFNA group. This difference is largely attributable to the longer bed rest and delayed weight-bearing in the PFNA group. The between-group HHS differences exceeded the commonly cited minimal clinically important difference (MCID) of approximately 8 points for hip arthroplasty. However, because rehabilitation protocols differed substantially, this clinical benefit cannot be attributed to the surgical approach alone. In our institution, the PFNA rehabilitation protocol prescribed bed-based exercises for the first 6–8 weeks, with standing and walking starting only after 6–8 weeks. This progressive weight-bearing regimen was designed according to the National Health Commission's Guidelines for the Diagnosis and Management of Geriatric Hip Fractures (2022 edition), which supports early weight-bearing but does not mandate immediate full weight-bearing, especially in osteoporotic patients (25). The delayed weight-bearing protocol after PFNA was not randomized and may reflect institutional tradition, concerns about osteoporotic bone, or surgeon preference. It is possible that some stable 31-A1 fractures could have been mobilized earlier. In recent years, our institution has adopted more individualized rehabilitation protocols, and most PFNA patients now begin early weight-bearing. Consequently, the PFNA group had a mean bedridden duration of nearly 53 days, which significantly delayed functional recovery compared with the DAA group, where patients were mobilized within one day postoperatively. Kumar et al. defined delayed weight-bearing after PFNA2 surgery as "after 4 weeks" in a prospective randomized controlled study, and found no significant difference in functional recovery at 24 weeks between the delayed weight-bearing group and the early weight-bearing group (26). In practical terms, this study compared DAA hemiarthroplasty followed by immediate mobilization with PFNA followed by delayed weight-bearing. Therefore, the functional outcome gap reflects both the surgical procedure and the rehabilitation strategy. In other words, DAA hemiarthroplasty provides patients with the opportunity for early rehabilitation, which may be an important reason for the differences in clinical outcomes, rather than the surgical differences directly causing the clinical differences. However, this does not contradict the purpose and conclusion of this study, namely that patients who chose DAA achieved faster recovery and better clinical outcomes.

Furthermore, our finding that HHS in the DAA group remained significantly higher even at 2 years (by 9.17 points) contrasts with a previous meta-analysis by Hongku et al., who reported no functional differences beyond 6 months (20). The discrepancy likely arises from differences in surgical approach (DAA vs. mostly posterolateral in previous studies) and the

much more aggressive early mobilization protocol in our DAA group. Previous studies have demonstrated that DAA offers faster rehabilitation for femoral neck fractures (14, 15, 27); our study extends this advantage to TF. Nevertheless, long-term outcomes beyond 2 years require further investigation. These differences should be interpreted in the context of both statistical and clinical significance. The HHS difference at 1 month was 14.96 points, and at 24 months was 9.17 points, both exceeding the MCID. Nevertheless, the rehabilitation confounder limits causal attribution.

Our work also documented and compared relevant intraoperative data. PFNA demonstrated significant advantages in terms of operative time and intraoperative blood loss. On average, each PFNA procedure lasted 40.05 min, which was 34.97 min shorter than that of hemiarthroplasty. Additionally, the blood loss per PFNA procedure was approximately 220.3 mL, 129.3 mL less than that of hemiarthroplasty. For patients at higher surgical risk, reducing operative time and blood loss can mitigate risks, particularly in elderly patients who often have multiple underlying conditions. This is one of the reasons why surgeons have traditionally preferred fracture reduction and internal fixation for trochanteric fractures. PFNA may be considered for patients at higher surgical risk but this was not directly tested in the present study and should be regarded as a clinical consideration rather than a study finding. Previous evidence indicates that DAA surgical procedures impose higher demands on surgeons (28). In our cohort, PFNA was associated with shorter operative time and less intraoperative blood loss than DAA hemiarthroplasty. However, surgical difficulty was not objectively measured in this study.

Regarding complications, the incidence rates of prosthesis loosening, periprosthetic fractures, and joint dislocation were all zero. The DAA group exhibited fewer cases of lower limb venous thrombosis, skin pressure sores, and pulmonary complications compared to the PFNA group. However, these differences were not statistically significant in this study, possibly due to the small sample size. Increasing the sample size might reveal more pronounced differences. The sample size of 54 patients per group was relatively small. *Post hoc* power analysis confirmed that the study was adequately powered for the observed HHS differences but underpowered for rare complications. Therefore, the numerically lower complication rates in the DAA group should not be interpreted as evidence that DAA reduces complications. Complication analyses should be regarded as exploratory. Larger multicenter studies with adequate power are needed to compare rare adverse events between DAA hemiarthroplasty and PFNA.

Regarding the imaging results, we primarily documented the healing status of the greater trochanter and the subsidence of the femoral component following joint replacement surgery. In the DAA group, at the final follow-up, the femoral stem exhibited an average subsidence of 2.7 mm compared to the initial postoperative measurement. Migliorini et al. reported an average prosthesis subsidence of 2.8 mm following THA (29), a finding closely aligned with our observations. Bornes et al. posited that prosthesis subsidence is primarily correlated with patients' body mass index (30). In our study, two patients demonstrated prosthesis subsidence exceeding 5 mm, with a maximum of 5.3 mm, which we attribute to body weight and osteoporosis.

Recent studies by Hernández-Pascual et al. have evaluated factors influencing outcomes after cephalomedullary nailing. Their 2025 study found that immediate partial weight bearing postoperatively could negatively affect short-term survival in TF with at least sufficient reduction (31). Their 2021 study showed that distal locking configuration did not influence long-term mobility or survival, though static locking had more minor complications (32). Their 2022 study introduced the GammaTScore for predicting early cut-out and consolidation time (33). These findings underscore the importance of standardized rehabilitation and implant-related factors when interpreting outcomes after PFNA.

In summary, we suggest that for elderly patients with TF, treatment options should be individualized rather than routinely confined to internal fixation. For instance, when patients express a preference for early functional recovery, are unable to tolerate prolonged immobilization, and are medically fit for longer surgical durations, hemiarthroplasty may be considered as a viable alternative, with the DAA approach potentially offering advantages in this context. Fatemeh Ghasemi et al. reported that quality of life following internal fixation of trochanteric fractures was suboptimal in several domains, particularly among elderly patients (34). These findings suggest that internal fixation may not always be the most suitable initial option for all elderly patients with TF, and patient-centered factors should be carefully weighed in surgical decision-making. The present study cannot determine whether the difference in functional recovery was caused by the arthroplasty itself, the DAA approach, the postoperative rehabilitation strategy, or a combination of these factors.

This study has several important limitations. First, despite the use of propensity score matching, the retrospective, non-randomized design is inherently subject to selection bias. Propensity score matching (PSM) can only balance observed covariates. Important unmeasured factors such as pre-injury functional status, frailty, cognitive function, bone mineral density, nutritional status, socioeconomic factors, and surgeon-specific decision-making were not included in the matching model. Second, the most significant confounder is the substantial difference in postoperative rehabilitation protocols between the two groups. The PFNA group followed a progressive weight-bearing regimen (6–8 weeks of bed-based exercises), which is more conservative than the immediate mobilization in the DAA group. This difference, rather than the surgical technique alone, likely contributed substantially to the observed functional outcome gap. Third, the sample size is relatively small, which may have limited statistical power to detect differences in rare complications. Fourth, the follow-up period was only 2 years, which is insufficient to evaluate long-term prosthesis survival or late-onset complications. Fifth, this was a single-center study performed by surgeons with substantial experience in DAA. The results may not be generalizable to centers with less DAA expertise or different learning curves. Sixth, we did not include other surgical approaches (e.g., posterolateral hemiarthroplasty) or total hip arthroplasty, so the comparative effectiveness of DAA vs. other arthroplasty techniques remains unknown. Therefore, our

conclusions should be considered hypothesis-generating and require validation in large-scale, prospective, randomized controlled trials.

5 Conclusion

In conclusion, in this propensity score-matched retrospective study, DAA hemiarthroplasty combined with early rehabilitation exercise was associated with faster early functional recovery compared with PFNA internal fixation in elderly patients with TF. However, PFNA offered advantages of shorter operative time, less blood loss, and lower surgical complexity. The study was underpowered for rare complications, and complication-related findings should be considered exploratory. Importantly, the two groups differed substantially in postoperative rehabilitation protocols, which may have contributed to the observed functional differences. Given the limitations inherent to the retrospective design and the single-center setting, these findings should be interpreted with caution. Individualized treatment decisions should balance patient preferences, bone quality, general health, and surgeon expertise. Prospective randomized trials with standardized rehabilitation protocols are needed to confirm the potential benefits of DAA hemiarthroplasty for this population.

Q11 Data availability statement

The raw data supporting the conclusions of this article will be made available by the authors, without undue reservation.

Q12 Ethics statement

The studies involving humans were approved by Ethics Committee of Jingjiang people's Hospital Affiliated to Yangzhou University. The studies were conducted in accordance with the local legislation and institutional requirements. Written informed consent for participation was not required from the participants or the participants' legal guardians/next of kin in accordance with the national legislation and institutional requirements. Written informed consent was obtained from the individual(s) for the publication of any potentially identifiable images or data included in this article.

Written informed consent for publication of identifiable images was obtained from all patients or their next of kin. All data were anonymized.

Q13 Author contributions

YJ: Data curation, Funding acquisition, Writing – original draft, Writing – review & editing. LX: Data curation, Writing – review & editing. HB: Conceptualization, Writing – review & editing. JH: Conceptualization, Writing – review & editing. ZW: Conceptualization, Data curation, Formal analysis, Funding acquisition, Project administration, Writing – review & editing.

Funding

The author(s) declared that financial support was received for Q14 this work and/or its publication. Our work is supported by the Jiangsu Traditional Chinese Medicine Science and Technology Development Plan Project (MS2021091), the Jingjiang People's Hospital Hospital-level Scientific Research Fund Project (JRY-KY-2023-016) and the Jingjiang People's Hospital Hospital-level Scientific Research Fund Project (JRY-KY-2026-022).

Acknowledgments

We thank the Radiology Department of the Jingjiang People's Hospital Affiliated to Yangzhou University for their technical support.

Conflict of interest Q15

The author(s) declared that this work was conducted in the absence of any commercial or financial relationships that could be construed as a potential conflict of interest.

Generative AI statement

The author(s) declared that generative AI was used in the creation of this manuscript. During the preparation of this manuscript, the authors used AI-assisted language refinement tools solely for grammar correction, spelling improvement, and fluency enhancement. No AI tools were used for content generation, data analysis, interpretation, or scientific conclusions. The authors have reviewed and edited the final content and take full responsibility for the integrity and accuracy of the work.

Any alternative text (alt text) provided alongside figures in this article has been generated by Frontiers with the support of artificial intelligence and reasonable efforts have been made to ensure accuracy, including review by the authors wherever possible. If you identify any issues, please contact us.

Publisher's note

All claims expressed in this article are solely those of the authors and do not necessarily represent those of their affiliated organizations, or those of the publisher, the editors and the reviewers. Any product that may be evaluated in this article, or claim that may be made by its manufacturer, is not guaranteed or endorsed by the publisher.

Supplementary material

The Supplementary Material for this article can be found online at: https://www.frontiersin.org/articles/10.3389/fsurg.2026.1954682/abstract#supplementary-material Q16

Q17

References

1. Lindskog DM, Baumgaertner MR. Unstable intertrochanteric hip fractures in the elderly. *J Am Acad Orthop Surg*. (2004) 12(3):179–90. doi: 10.5435/00124635-200405000-00006

2. Lu-Yao GL, Keller RB, Littenberg B, Wennberg JE. Outcomes after displaced fractures of the femoral neck. A meta-analysis of one hundred and six published reports. *J Bone Joint Surg Am*. (1994) 76(1):15–25. doi: 10.2106/00004623-199401000-00003

3. Wang H, Yang W, Ding K, Zhu Y, Zhang Y, Ren C, et al. Biomechanical study on the stability and strain conduction of intertrochanteric fracture fixed with proximal femoral nail antirotation versus triangular supporting intramedullary nail. *Int Orthop*. (2022) 46(2):341–50. doi: 10.1007/s00264-021-05250-8

4. Jones HW, Johnston P, Parker M. Are short femoral nails superior to the sliding hip screw? A meta-analysis of 24 studies involving 3,279 fractures. *Int Orthop*. (2006) 30(2):69–78. doi: 10.1007/s00264-005-0028-0

5. Chu X, Liu F, Huang J, Chen L, Li J, Tong P. Good short-term outcome of arthroplasty with Wagner SL implants for unstable intertrochanteric osteoporotic fractures. *J Arthroplasty*. (2014) 29(3):605–8. doi: 10.1016/j.arth.2013.07.029

6. Liu L, Sun Y, Wang L, Gao Q, Li A, Wang J, et al. Total hip arthroplasty for intertrochanteric fracture fixation failure. *Eur J Med Res*. (2019) 24(1):39. doi: 10.1186/s40001-019-0398-1

7. Lee KH, Lee DH, Noh JH, Kim YV. Is rigid fixation of the greater trochanter necessary for arthroplasty of intertrochanteric fractures? *Orthop Traumatol Surg Res*. (2019) 105(1):41–5. doi: 10.1016/j.otsr.2018.09.015

8. Lee Y-K, Kim JT, Alkitaini AA, Kim K-C, Ha Y-C, Koo K-H. Conversion hip arthroplasty in failed fixation of intertrochanteric fracture: a propensity score matching study. *J Arthroplasty*. (2017) 32(5):1593–8. doi: 10.1016/j.arth.2016.12.018

9. Gjertsen JE. Should total hip arthroplasty be used for hip fracture? *N Engl J Med*. (2019) 381(23):2261–2. doi: 10.1056/NEJMe1913800

10. Waddell JP, Morton J, Schemitsch EH. The role of total hip replacement in intertrochanteric fractures of the femur. *Clin Orthop Relat Res*. (2004) 429:49–53. doi: 10.1097/01.blo.0000150113.11866.48

11. Barrett WP, Turner SE, Leopold JP. Prospective randomized study of direct anterior vs postero-lateral approach for total hip arthroplasty. *J Arthroplasty*. (2013) 28(9):1634–8. doi: 10.1016/j.arth.2013.01.034

12. Christensen CP, Jacobs CA. Comparison of patient function during the first six weeks after direct anterior or posterior total hip arthroplasty (THA): a randomized study. *J Arthroplasty*. (2015) 30(9 Suppl):94–7. doi: 10.1016/j.arth.2014.12.038

13. Barrett WP, Turner SE, Murphy JA, Flener JL, Alton TB. Prospective, randomized study of direct anterior approach vs posterolateral approach total hip arthroplasty: a concise 5-year follow-up evaluation. *J Arthroplasty*. (2019) 34(6):1139–42. doi: 10.1016/j.arth.2019.01.060

14. Di Martino A, Brunello M, Rossomando V, Pederiva D, Schilardi F, Stefanini N, et al. Aesthetic results, functional outcome and radiographic analysis in THA by direct anterior, bikini and postero-lateral approach: is it worth the hassle? *J Clin Med*. (2023) 12(3):1072. doi: 10.3390/jcm12031072

15. Shen J, Ji R, Yao S, Ruan P, Zhu Z, Wang X, et al. Direct anterior approach provides superior prosthesis adaptability in the early postoperative period of total hip arthroplasty. *Orthop Surg*. (2023) 15(3):679–86. doi: 10.1111/os.13640

16. Marsh JL, Slongo TF, Agel J, Broderick JS, Creevey W, DeCoster TA, et al. Fracture and dislocation classification compendium—2007: Orthopaedic Trauma Association classification, database and outcomes committee. *J Orthop Trauma*. (2007) 21(10 Suppl):S1–6. doi: 10.1097/00005131-200711101-00001

17. Fogagnolo F, Kfuri M Jr, Paccola CA. Intramedullary fixation of pertrochanteric hip fractures with the short AO-ASIF proximal femoral nail. *Arch Orthop Trauma Surg*. (2004) 124(1):31–7. doi: 10.1007/s00402-003-0586-9

18. Engh CA, Bobyn JD, Glassman AH. Porous-coated hip replacement. The factors governing bone ingrowth, stress shielding, and clinical results. *J Bone Joint Surg Br*. (1987) 69-B(1):45–55. doi: 10.1302/0301-620X.69B1.3818732

19. Yuan BJ, Abdel MP, Cross WW, Berry DJ. Hip arthroplasty after surgical treatment of intertrochanteric hip fractures. *J Arthroplasty*. (2017) 32(11):3438–44. doi: 10.1016/j.arth.2017.06.032

20. Hongku N, Woratanarat P, Nitiwarangkul L, Rattanasiri S, Thakkinstian A. Fracture fixation versus hemiarthroplasty for unstable intertrochanteric fractures in elderly patients: a systematic review and network meta-analysis of randomized controlled trials. *Orthop Traumatol Surg Res*. (2022) 108(1):102838. doi: 10.1016/j.otsr.2021.102838

21. Bretherton CP, Parker MJ. Femoral medialization, fixation failures, and functional outcome in trochanteric hip fractures treated with either a sliding hip screw or an intramedullary nail from within a randomized trial. *J Orthop Trauma*. (2016) 30(12):642–6. doi: 10.1097/BOT.0000000000000689

22. Gilat R, Lubovsky O, Atoun E, Debi R, Cohen O, Weil YA. Proximal femoral shortening after cephalomedullary nail insertion for intertrochanteric fractures. *J Orthop Trauma*. (2017) 31(6):311–5. doi: 10.1097/BOT.0000000000000835

23. He M, Liu J, Deng X, Zhang X. The postoperative prognosis of older intertrochanteric fracture patients as evaluated by the Chang reduction quality criteria. *BMC Geriatr*. (2022) 22(1):928. doi: 10.1186/s12877-022-03641-z

24. Yu J, Zhang C, Li L, Kwong JSW, Xue L, Zeng X, et al. Internal fixation treatments for intertrochanteric fracture: a systematic review and meta-analysis of randomized evidence. *Sci Rep*. (2016) 5:18195. doi: 10.1038/srep18195

25. National Health Commission of the People's Republic of China. *Guideline for the Diagnosis and Management of Geriatric Hip Fractures*. 2022 ed. Beijing: People's Medical Publishing House (2022).

26. Kumar A, Jhariya R, Kumar S, Pandey R, Yadav S. Clinical-radiological outcomes of early versus delayed weight-bearing following proximal femoral nail antirotation 2 fixation for intertrochanteric fractures: a prospective randomized comparative study. *Cureus*. (2026) 18(1):e101530. doi: 10.7759/cureus.101530

27. Bon G, Kacem EB, Lepretre PM, Weissland T, Mertl P, Dehl M, et al. Does the direct anterior approach allow earlier recovery of walking following total hip arthroplasty? A randomized prospective trial using accelerometry. *Orthop Traumatol Surg Res*. (2019) 105(3):445–52. doi: 10.1016/j.otsr.2019.02.008

28. Stone AH, Sibia US, Atkinson R, Turner TR, King PJ. Evaluation of the learning curve when transitioning from posterolateral to direct anterior hip arthroplasty: a consecutive series of 1000 cases. *J Arthroplasty*. (2018) 33(8):2530–4. doi: 10.1016/j.arth.2018.02.086

29. Migliorini F, Maffulli N, Pilone M, Kämmer D, Hofmann UK, Nobili A, et al. Subsidence of the Corail stem in total hip arthroplasty: no influence of bony contact. *J Orthop Traumatol*. (2024) 25(1):53. doi: 10.1186/s10195-024-00794-y

30. Bornes TD, Radomski LR, Bonello J-P, Mortensen-Truscott L, Safir OA, Gross AE, et al. Subsidence of a single-taper femoral stem in primary total hip arthroplasty: characterization, associated factors, and sequelae. *J Arthroplasty*. (2023) 38(7S):S174–8. doi: 10.1016/j.arth.2023.04.026

31. Hernández-Pascual C, Santos-Sánchez JÁ, Hernández-Rodríguez J, Silva-Viamonte CF, Pablos-Hernández C, Alonso-Rodríguez P, et al. Partial weight bearing and long-term survival outcomes in extracapsular hip fractures treated with trochanteric Gamma3 nails. *BMC Musculoskelet Disord*. (2025) 26(1):129. doi: 10.1186/s12891-024-08043-3

32. Hernández-Pascual C, Santos-Sánchez JÁ, García-González JM, Silva-Viamonte CF, Pablos-Hernández C, Ramos-Pascua L, et al. Long-term outcomes of distal locking in extracapsular fractures treated with trochanteric Gamma3 nails. *J Orthop Traumatol*. (2021) 22(1):48. doi: 10.1186/s10195-021-00609-4

33. Hernández-Pascual C, Santos-Sánchez JÁ, Hernández-Rodríguez J, Silva-Viamonte CF, Pablos-Hernández C, Villanueva-Martínez M, et al. New prognostic factors in operated extracapsular hip fractures: infection and GammaTScore. *Int J Environ Res Public Health*. (2022) 19(18):11680. doi: 10.3390/ijerph191811680

34. Ghasemi F, Esmaeilnejad-Ganji SM, Manafi Rasi A, Afzal S, Baroutkoub M, Tavassoli M. Evaluation of quality of life and associated factors in patients with intertrochanteric femoral fracture. *PLoS One*. (2023) 18(11):e0293686. doi: 10.1371/journal.pone.0293686
